# Cyanide Removal by ZnTiO_3_/TiO_2_/H_2_O_2_/UVB System: A Theoretical-Experimental Approach

**DOI:** 10.3390/ijms242216446

**Published:** 2023-11-17

**Authors:** Ximena Jaramillo-Fierro, John Ramón, Eduardo Valarezo

**Affiliations:** 1 Departamento de Química, Facultad de Ciencias Exactas y Naturales, Universidad Técnica Particular de Loja, San Cayetano Alto, Loja 1101608, Ecuador; bevalarezo@utpl.edu.ec; 2 Ingeniería Química, Facultad de Ciencias Exactas y Naturales, Universidad Técnica Particular de Loja, San Cayetano Alto, Loja 1101608, Ecuador; jbramon2@utpl.edu.ec

**Keywords:** cyanide, adsorption, photocatalysis, toxicity, DFT calculations

## Abstract

Cyanide is a highly toxic substance present in wastewater from various industries. This study investigates the removal of cyanide species (CS) from aqueous solutions using the ZnTiO_3_/TiO_2_/H_2_O_2_/UVB system. ZnTiO_3_/TiO_2_ nanoparticles synthesized by the sol-gel method were characterized by powder X-ray diffractometry (XRD), scanning electron microscopy (SEM), and energy-dispersive X-ray spectroscopy (EDS). The adsorption capacity of nanoparticles was tested by varying the pH of the solution, adsorbent concentration, and contact time. The adsorption of CS on ZnTiO_3_ and TiO_2_ surfaces was verified by Density Functional Theory (DFT) calculations. Photocatalytic experiments were achieved under UVB irradiation (λ = 310 nm). The response surface methodology (RSM) was used to optimize the CS removal efficiency. The detoxification effect was evaluated by acute toxicity tests with brine shrimp. The theoretical results show that the adsorption of CS is energetically more favorable on the ZnTiO_3_ surface than on the TiO_2_ surface. The experimental results show that the system consisting of ZnTiO_3_/TiO_2_ (200 mg L^−1^), H_2_O_2_ (0.1%), and UVB light removes 99% of CS from aqueous solutions after 60 min and reduces the mortality of nauplii in 90% after 90 min. This system was reused in five consecutive cycles with a total loss of efficiency of 30%.

## 1. Introduction

The presence of contaminants in wastewater represents a significant and potentially hazardous risk to both the environment and public health. Therefore, wastewater treatment is a principal concern within our society [1]. Cyanide is a prevalent contaminant found in wastewater from gold mines and various industrial sectors [2]. Cyanides are highly toxic chemical compounds that can cause severe harm to both human health and the environment [3,4]. Cyanide compounds exist in two primary forms in the environment: As unbound entities such as the cyanide ion (CN^−^) and hydrogen cyanide (HCN), and as water-soluble inorganic salts like sodium cyanide (NaCN) and potassium cyanide (KCN) [5]. All forms of cyanide can be lethal at high exposure levels, with free cyanide (HCN + CN^−^) being the most toxic [6]. Industrial effluent discharge is the primary source of cyanide complexes entering the environment, as these complexes form through the combination of cyanide ions with metal ions [7]. Given the potential hazards associated with cyanide complexes, it is crucial to regulate and remediate cyanide-contaminated water before releasing it into the environment [8,9].

Traditionally, the removal of cyanide and its associated chemicals during industrial wastewater treatment relies on biological and physicochemical treatment methods [10,11]. Biological treatment is cost effective and environmentally friendly, but it has limitations when dealing with high cyanide concentrations due to the slow detoxification rate of cyanide [12]. Physicochemical methods such as filtration, precipitation, coagulation, reverse osmosis, electrolytic oxidation, acidification, flotation, thermal hydrolysis, alkaline chlorination, and the AVR (acidification–volatilization–reabsorption) process can eliminate or transform cyanide into less harmful forms [13,14]. However, some of these methods are costly, involve hazardous chemical reagents, require specialized equipment, demand continuous maintenance, and include additional detoxification stages [15,16].

Alternatively, adsorption and advanced oxidation processes (AOPs) have emerged as promising techniques for cyanide removal due to their high efficiency, simplicity in design, ease of operation, and low operating costs [17,18,19]. The adsorption process can occur through various mechanisms, including electrostatic interaction, ion exchange, and complexation [20]. However, adsorption alone only transfers the contaminant from one medium to another without complete removal. Complete degradation of the adsorbed pollutant can be achieved through a series of oxidation-reduction reactions inherent to AOPs [21,22,23].

Various AOPs have been used for the treatment of cyanide-contaminated waters, including the Fenton and Photo-Fenton reactions, ozonation (O_3_), ultraviolet (UV) photolysis, UV/O_3_, UV/H_2_O_2_, and H_2_O_2_-based catalytic reactions [24]. H_2_O_2_ is a high-value multifunctional oxidant that is widely applied in a wide range of industries, such as chemical synthesis, energy-related applications, medical disinfection, and wastewater treatment [25]. Hydrogen peroxide (H_2_O_2_) has attracted attention because it can address the limitations of conventional treatments and other AOPs. H_2_O_2_ can oxidize cyanide without generating toxic byproducts and enhances the catalytic degradation of cyanide. However, practical applicability may be limited when dealing with large wastewater volumes due to the considerable time and H_2_O_2_ quantity required. Therefore, additional components, such as photocatalysts and UV photolysis, are essential to enhance cyanide removal efficiency [12].

Adsorbing the contaminant onto the photocatalyst before photodegradation is crucial for enhancing the speed and effectiveness of contaminant removal in aqueous solutions. This process concentrates the contaminant on the surface of the photocatalyst, where it can undergo more efficient degradation through redox reactions involving radicals generated during photodegradation [26]. Photocatalysis in aqueous systems generates reactive oxygen species (ROS), powerful oxidizing agents capable of breaking down stubborn compounds [5]. Titanium dioxide (TiO_2_) is a widely used semiconducting material in photocatalytic applications due to its stability, cost-effectiveness, low toxicity, and environmentally friendly nature [27,28,29]. However, the use of TiO_2_ is restricted to ultraviolet (UV) wavelengths due to its specific bandgap energy requirement (E_g_ = 3.2 eV) [30,31,32]. Another limitation of TiO_2_ is its tendency for rapid electron-hole recombination, reducing ROS formation and photocatalytic efficiency [33].

To address these limitations and enhance the photocatalytic process, various surface modifications of TiO_2_ have been proposed. One such modification involves coupling TiO_2_ with other semiconductors that have different redox energy levels for their respective conduction (CB) and valence (VB) bands. This coupling improves charge separation, prolongs charge carrier lifetimes, and enhances interfacial charge transfer to adsorbed substrates [34,35]. Numerous semiconductors, including cadmium sulfide (CdS), copper(II) oxide (CuO), iron(III) oxide (Fe_2_O_3_), magnesium oxide (MgO), molybdenum trioxide (MoO_3_), tin(IV) oxide (SnO_2_), silicon dioxide (SiO_2_), tungsten(VI) oxide (WO_3_), zirconium dioxide (ZrO_2_), and zinc oxide (ZnO), have been considered for coupling with TiO_2_ [36,37,38,39]. The properties and compatibility of the coupled semiconductor significantly impact the surface charge of the material and, consequently, its photocatalytic capacity [40].

The coupled semiconductor ZnTiO_3_/TiO_2_, which integrates zinc titanate (ZnTiO_3_) with TiO_2_, has been explored for the removal of various pollutants in wastewater due to its molecular properties and adsorption and photocatalysis characteristics [41,42]. This hybrid semiconductor exhibits superior physical and chemical properties compared to individual components, primarily due to alterations in their electronic states. In fact, the combination of ZnTiO_3_ and TiO_2_ in the ZnTiO_3_/TiO_2_ photocatalyst takes advantage of the complementary properties of both materials [43,44]. While TiO_2_ is effective in generating reactive oxygen species in the presence of ultraviolet light, ZnTiO_3_ improves visible light absorption and charge carrier mobility, increasing the overall efficiency of photocatalysis [45,46]. Furthermore, the presence of ZnTiO_3_ can provide additional adsorption sites for various contaminants, facilitating their interaction with the photocatalyst and promoting their efficient removal from wastewater. Based on these properties, it is suggested that in the specific case of cyanide removal, ZnTiO_3_/TiO_2_ could certainly exhibit high efficiency.

The adsorption and photodegradation capabilities of materials are influenced by numerous factors, including material properties and operational conditions [47]. A comprehensive exploration of these parameters provides valuable insights for designing materials and processes to efficiently remove specific contaminants [48,49]. While the literature contains numerous reports on materials for removing NaCN, KCN, and cyanide complexes, to our knowledge, no prior studies have addressed the adsorption and photodegradation of cyanide species using the ZnTiO_3_/TiO_2_ hybrid semiconductor in a ZnTiO_3_/TiO_2_/H_2_O_2_/UVB system. Consequently, the behavior of these semiconductors in adsorption and photodegradation processes involving cyanide species remains unclear. Therefore, this study aims to evaluate the effectiveness of the ZnTiO_3_/TiO_2_ H_2_O_2_/UVB system in removing total cyanide from aqueous solutions. 

In order to investigate cyanide removal by adsorption and photodegradation processes, batch experiments were carried out. These batch experiments consisted of the preparation of samples of aqueous solutions containing known concentrations of cyanide, which were subjected to controlled treatment conditions using the ZnTiO_3_/TiO_2_ H_2_O_2_/UVB system. In each experiment, a precise amount of the cyanide solution was added to the reactor system, followed by a period of stirring to allow interaction between ZnTiO_3_/TiO_2_/H_2_O_2_ and the cyanide present in the solution. Subsequently, irradiation with ultraviolet type B (UVB) light was applied to the sample. During the course of the experiments, periodic samples of the treated solution were taken to determine the residual cyanide concentration. This was achieved through spectrophotometry, which made it possible to precisely quantify the amount of cyanide present in the solution at different times.

The uniqueness of this comparative study lies in its demonstration of efficient cyanide species removal from aqueous systems with minimal H_2_O_2_ consumption, thanks to the synergistic combination of adsorption and photocatalysis processes in the ZnTiO_3_/TiO_2_ semiconductor. Finally, the materials synthesized in this study hold promise as an innovative environmental technology, offering operability and reusability, and presenting a sustainable alternative with significant potential for effluent treatment applications.

## 2. Results

Table 1 shows a summary of the mathematical expressions used in this study for data analysis.

### 2.1. Characterization of the Nanoparticles

#### 2.1.1. XRD Analysis

**Figure 1:**
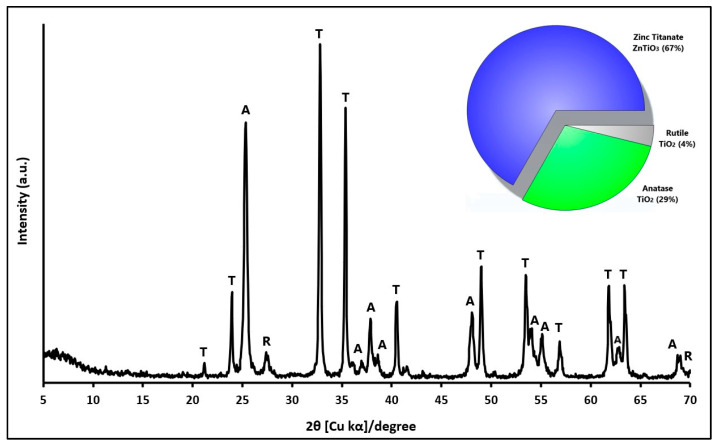
XRD patterns of ZnTiO_3_/TiO_2_ nanoparticles. T: Zinc Titanate (ZnTiO_3_), A: Anatase (TiO_2∙A_), and R: Rutile (TiO_2∙R_).

The X-ray diffraction patterns presented in Figure 1 illustrate the structural composition of the ZnTiO_3_/TiO_2_ semiconductor. This hybrid semiconductor was synthesized at a temperature of 500 °C, using a TiO_2_:ZnO molar ratio of 3:1. The XRD analysis reveals that the hybrid semiconductor is predominantly composed of two distinct phases: ZnTiO_3_ (T), accounting for approximately 67% of the material, and TiO_2_ in the anatase phase (A), constituting approximately 29% of the composition. Additionally, a minor fraction of approximately 4% consists of TiO_2_ in the rutile phase (R). The crystal structure of the ZnTiO_3_ phase was identified as rhombohedral, with unit cell parameters a = b = 5.08 Å and c = 13.93 Å. This crystal structure corresponds to space group R-3(148) as per the standard COD card No. 00-026-1500. On the other hand, the TiO_2_ phase in the anatase form exhibited a tetragonal crystal structure with unit cell parameters a = b = 3.79 Å and c = 9.51 Å, consistent with space group I41/amd(141) based on the standard COD card No. 96-901-5930. Trace amounts of TiO_2_ in the rutile phase were also detected, characterized by a tetragonal crystal structure with unit cell parameters a = b = 4.59 Å and c = 2.96 Å. This phase was attributed to space group P42/mnm(136) using the standard COD card No. 96-900-7532.

To determine the crystal dimensions of the ZnTiO_3_/TiO_2_ nanoparticles was employed the Scherrer equation [50], as outlined in Equation (1) (see Table 1). This calculation considered the most prominent peaks corresponding to the respective crystallographic phases. The results indicated an average crystallite size of approximately 34.16 (±2.91) nm for ZnTiO_3_ and approximately 23.19 (±3.07) nm for TiO_2_ in the anatase phase (TiO_2-A_).

#### 2.1.2. SSA and pH_PZC_ Analysis

In this study, an analysis of specific surface area (SSA) using the single-point BET (Brunauer–Emmet–Teller) method and a point of zero charge (pH_PZC_) was conducted to gain insights into the surface properties and charge characteristics of the ZnTiO_3_/TiO_2_ nanoparticles. The results obtained in the BET analysis revealed that the ambient pressure recorded was 596 mmHg, while the saturation pressure reached 611 mmHg. Furthermore, the sectional area of the sample was determined to be 0.162 nm^2^. Regarding the volume of an adsorbed monolayer, a value of 21.26 cm^3^/g was obtained. Finally, the BET surface area of the sample was calculated to be 92.53 m^2^/g. The plot of the TCD signal (a.u.) versus time is shown in Figure S1.

**Figure 2:**
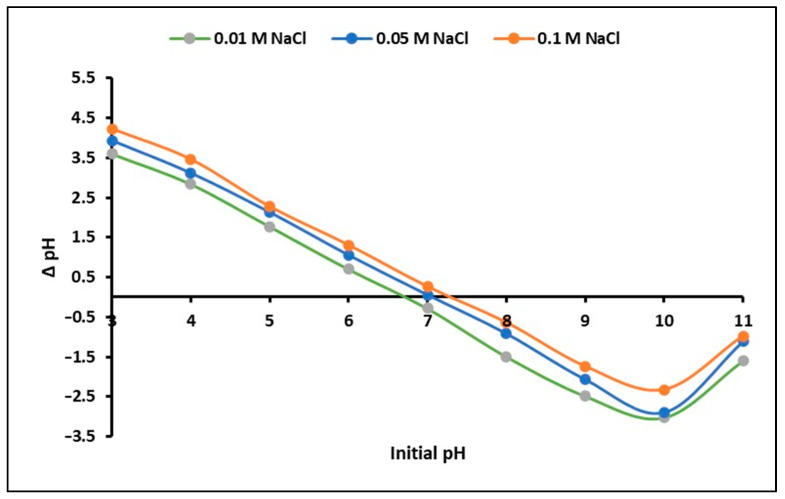
Diagram to determine the point of zero charge of ZnTiO_3_/TiO_2_ nanoparticles.

On the other hand, regarding the point of zero charge of the nanoparticles, the results obtained in this study are presented in Figure 2. This parameter denotes the pH at which the net surface charge of the solid becomes neutral under specific conditions [51]. In this study, the pH_PZC_ for the ZnTiO_3_/TiO_2_ nanoparticles was found to be approximately 7.0. This means that at a pH lower than the pH_PZC_ (pH < pH_PZC_ = 7.0), the nanoparticles exhibit a positively charged surface. Conversely, at pH levels higher than the pH_PZC_ (pH > pH_PZC_ = 7.0), the nanoparticles display a negatively charged surface.

These results provide crucial information about the physicochemical properties of the studied material, which is relevant to understanding its behavior and potential application in various scientific and technological areas.

#### 2.1.3. SEM and EDS Analysis

**Figure 3:**
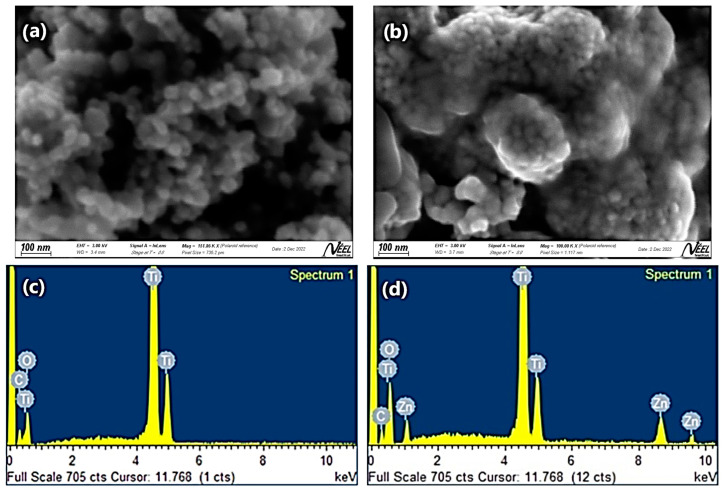
SEM image of (**a**) TiO_2_ and (**b**) ZnTiO_3_/TiO_2_ nanoparticles, and EDS spectrum of (**c**) TiO_2_ and (**d**) ZnTiO_3_/TiO_2_ nanoparticles.

In this study, morphological and dimensional characterization of ZnTiO_3_/TiO_2_ nanoparticles was carried out by scanning electron microscopy (SEM) and image analysis. Additionally, the characterization of the TiO_2_ nanoparticles was included to establish a comparison with the ZnTiO_3_/TiO_2_ nanoparticles. The SEM images (Figure 3a,b) revealed a nearly spherical morphology and high nanoparticle density in both semiconductors. However, significant differences in the shape and tendency to form clusters were observed between TiO_2_ and ZnTiO_3_/TiO_2_ nanoparticles. These observations are consistent with previous reports from other researchers [52].

The average size of the nanoparticles was determined using ImageJ2 software version 1.54 g, a widely accepted tool for scientific image processing and analysis [53,54]. From this analysis, values of 37.6 ± 7.2 nm for TiO_2_ and 27.4 ± 4.9 nm for ZnTiO_3_/TiO_2_ were obtained. The complete particle size distribution for TiO_2_ and ZnTiO_3_/TiO_2_ is shown in Figures S2 and S3 of the supplementary material, respectively. These results agree with the data obtained from the X-ray diffraction analysis (Figure 1) for ZnTiO_3_/TiO_2_ and with previous reports by other researchers for TiO_2_ [55], supporting the reliability of the measurements.

Furthermore, EDS analysis was used to determine the elemental composition of the TiO_2_ and ZnTiO_3_/TiO_2_ nanoparticles (Figure 3c,d). The results of the elemental analysis are summarized in Table 2. 

### 2.2. Optimization of Cyanide Removal Process

**Figure 4:**
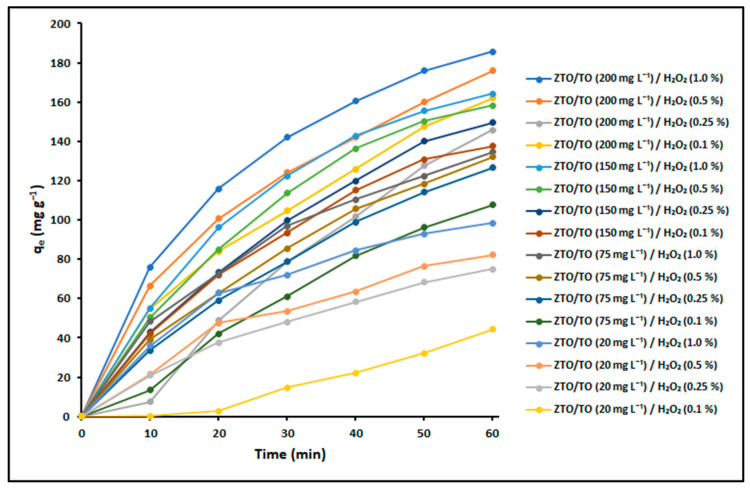
Cyanide removal capacity as a function of reaction system composition. Cyanide concentration = 100 mg L^−1^, Solution pH = 10, Temperature = 20 °C, and n = 3.

In this study, we investigated the effect of varying the ZnTiO_3_/TiO_2_:H_2_O_2_ ratio on the removal capacity (q_e_) of cyanide species (HCN and CN^–^), measured in milligrams per gram (mg g^−1^). The experimental results, obtained from 16 different combinations of ZnTiO_3_/TiO_2_ (ZTO/TO) and H_2_O_2_, are displayed in Figure 4. It is important to note that at pH = 10, the contribution of hydrogen cyanide (HCN) to the total cyanide concentration is minimal, since the dissociation of HCN into CN^–^ and H^+^ is favored, resulting in a greater proportion of CN^–^ compared to HCN. Therefore, in the test performed at pH = 10, the predominant form of cyanide is expected to be the cyanide ion (CN^−^) in its free form.

To further assess the statistical significance of these variations, an analysis of variance (ANOVA) was conducted, and the results are summarized in Table 3. This table provides information on the significance of the observed differences observed in q_e_ values among the various reaction system compositions. Specifically, values within the same column marked with distinct letters (a–k) indicate significant differences between the corresponding data points. These differences are statistically significant at a significant level (*p* < 0.01).

The remarkable effectiveness of H_2_O_2_ in removing cyanide species from aqueous systems is widely recognized. Nevertheless, the utilization of great quantities of H_2_O_2_ for extensive-scale wastewater treatment is impractical. In light of this consideration, a reaction system comprising 200 mg L^−1^ of ZnTiO_3_/TiO_2_ and 0.1% H_2_O_2_ was chosen to perform the removal experiments.

### 2.3. Adsorption Studies

#### 2.3.1. Effect of pH on Cyanide Adsorption

**Figure 5:**
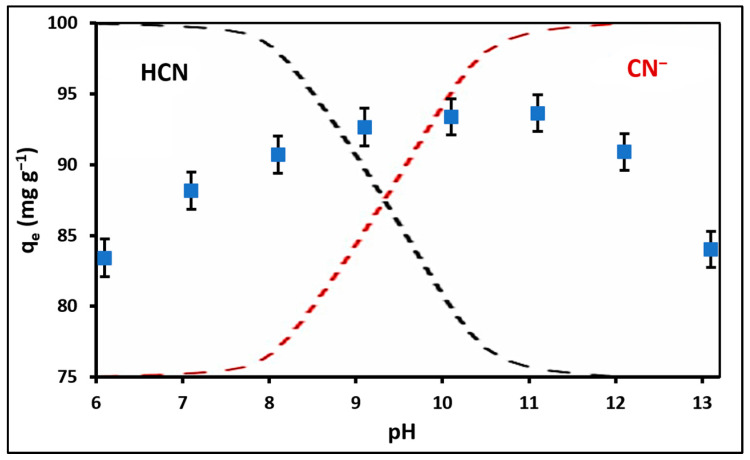
Effect of solution pH on the cyanide adsorption capacity of ZnTiO_3_/TiO_2_ nanoparticles. $HCN↔{CN}^{-}+H^{+}$ (pKa = 9.4).

Understanding the pH-dependent behavior of adsorption is fundamental for explaining the principal mechanisms governing the interaction between cyanide species and nanoparticle surfaces. Figure 5 shows the cyanide speciation diagram as a function of pH in the background [56]. Furthermore, Figure 5 illustrates the results of the cyanide adsorption experiment with respect to the pH levels of the solutions. The combined adsorption capacity for total cyanide (HCN + CN^−^) showed minimal variation within the pH range of 9–11 for ZnTiO_3_/TiO_2_ nanoparticles (pH_PZC_ = 7.0). Consequently, in our study, removal experiments were performed at pH 10 to explore the behavior of cyanide species in a region where both HCN and CN^−^ are present, with the latter being in greater proportion.

#### 2.3.2. Maximum Cyanide Adsorption Capacity

**Figure 6:**
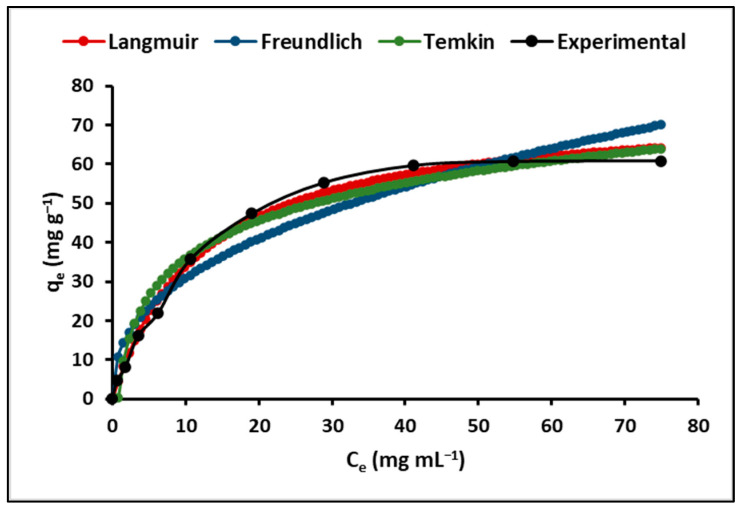
Cyanide adsorption isotherms of ZnTiO_3_/TiO_2_ nanoparticles.

Figure 6 illustrates the cyanide adsorption isotherms for ZnTiO_3_/TiO_2_ nanoparticles. This figure clearly demonstrates that, for the nanoparticles, the Langmuir model offers a more suitable representation of the adsorption process when compared to the Freundlich and Temkin models.

The estimated constants for the Langmuir, Freundlich, and Temkin models at absolute temperatures of 293.15 K, 298.15 K, and 303.15 K are displayed in Table 4. In this table, it can be observed that the values of the separation factor R_L_, also known as the equilibrium parameter, fall within the range of 0 to 1. Similarly, the coefficient “n”, which signifies the adsorption intensity, falls within the range of 1 to 10. As a result, it is reasonable to infer that the adsorption of cyanide species on the surface of ZnTiO_3_/TiO_2_ nanoparticles was found to be favorable and satisfactory.

#### 2.3.3. Adsorption Thermodynamics

**Figure 7:**
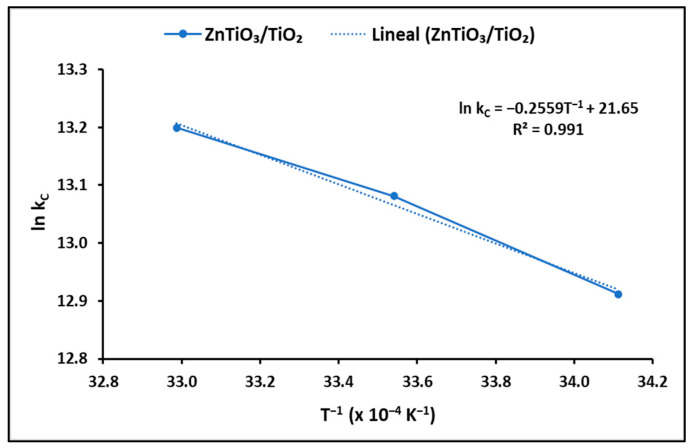
Thermodynamic study of cyanide adsorption onto ZnTiO_3_/TiO_2_ nanoparticles.

Thermodynamic parameters offer insights into the spontaneity and feasibility of a process. To ascertain these parameters, specifically the Gibbs free energy change (∆G°), enthalpy change (∆H°), and surface entropy change (∆S°), the equilibrium constant was determined at various temperatures, as depicted in Figure 7. 

The outcomes regarding the thermodynamic parameters are outlined in Table 5. ∆G° represents the degree of spontaneity in the process, with negative values signifying a higher degree of adsorption favorability. Correspondingly, positive ΔH° values indicate that the process is endothermic, while positive ΔS° values suggest an increase in randomness at the solution–solid interface during the adsorption process.

#### 2.3.4. Kinetic of Cyanide Adsorption

**Figure 8:**
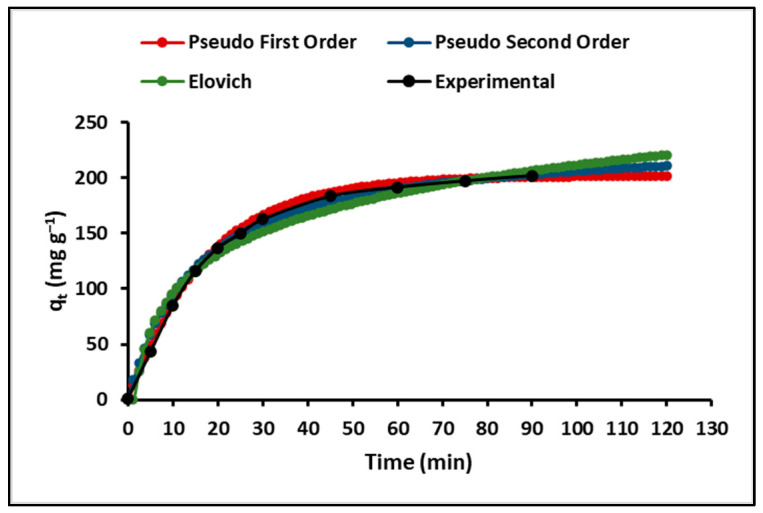
Adsorption kinetics of cyanide onto ZnTiO_3_/TiO_2_ nanoparticles.

The kinetics of the cyanide adsorption process onto ZnTiO_3_/TiO_2_ nanoparticles were investigated in this study, as understanding the time-dependent rate of adsorption is fundamental for the design and assessment of adsorbents in adsorption processes. To characterize the kinetics, three models were employed: the pseudo-first-order (Lagergren) model, the pseudo-second-order (Ho) model, and the Elovich model. As displayed in Figure 8, these models exhibit an initial rapid adsorption phase followed by a plateau phase. Table 4 reveals that the correlation coefficient of the pseudo-second-order model exceeds that of the pseudo-first-order and Elovich models.

**Figure 9:**
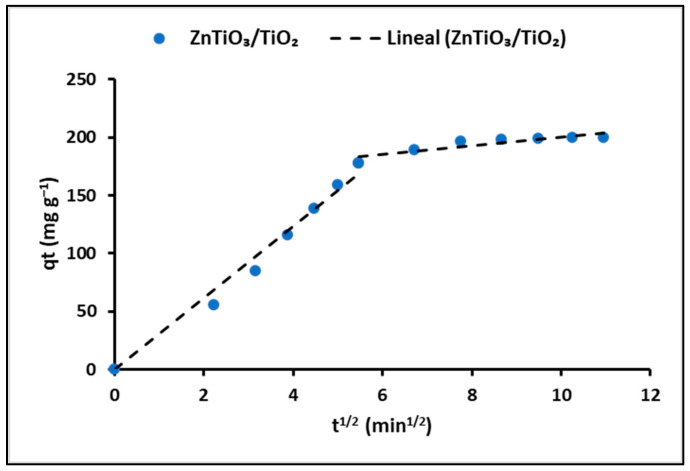
Intra-particle diffusion plots for cyanide removal by ZnTiO_3_/TiO_2_ nanoparticles.

The intraparticle diffusion model was used to elucidate the adsorption rate and the mechanism of cyanide species migration onto the nanoparticles. Figure 9 illustrates the alterations in the q_t_ (mg g^−1^) curves concerning time (t^1/2^) for the ZnTiO_3_/TiO_2_ nanoparticles.

Table 6 shows the total cyanide adsorption kinetic parameters calculated in this investigation for the ZnTiO_3_/TiO_2_ nanoparticles.

Finally, after the adsorption experiments, the materials with adsorbed cyanide were subjected to a leaching process. After the leaching period, an analysis was performed to determine the presence of cyanide in the aqueous solution. However, the results revealed that no cyanide leaching was detected under any of the conditions tested (pH 4, 7, and 10) after the leaching time of 24 h. Based on these findings, it was concluded that the adsorption materials used in the test demonstrated high retention of the adsorbed cyanide. The absence of cyanide leaching, even under stirring conditions in water at pH 4, 7, and 10, indicates that cyanide adsorption on the materials was strong and stable.

#### 2.3.5. DFT Study of the Cyanide Adsorption onto ZnTiO_3_/TiO_2_ Nanoparticles

**Figure 10:**
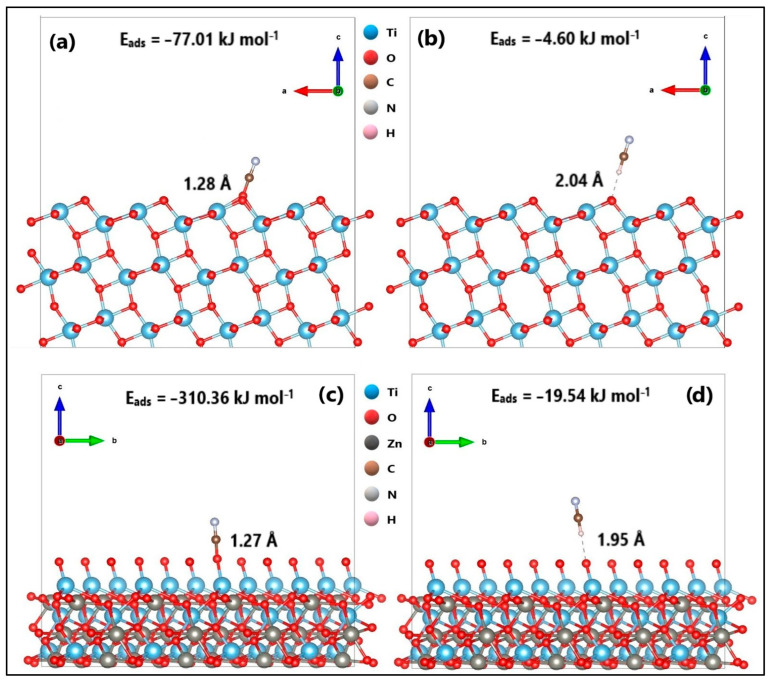
Cyanide species adsorbed on the surfaces 101 of the photocatalysts. (**a**) CN-TiO_2_, (**b**) HCN-TiO_2_, (**c**) CN-ZnTiO_3_, and (**d**) HCN-ZnTiO_3_.

In a previous investigation [57], the optimized structure of ZnTiO_3_ was represented by a hexagonal Bravais lattice with space group R-3(148). Similarly, the optimized structure of TiO_2_ in its anatase phase was represented by a tetragonal Bravais lattice with I41/amd(141) space group. In this study, the adsorption energy of cyanide species CN^−^ and HCN on the surfaces (101) of TiO_2_ and ZnTiO_3_ was evaluated using the Vienna Ab initio Simulation Package (VASP) code. Figure 10 provides information on the interatomic distances between the cyanide species and the semiconductor surfaces, as well as the corresponding adsorption energies (E_ads_).

To verify the chemical interactions between cyanide species (CN^−^ and HCN) and the semiconductor materials (TiO_2_ and ZnTiO_3_), a population analysis of CN-TiO_2_, HCN-TiO_2_, CN-ZnTiO_3_, and HCN-ZnTiO_3_ interactions was performed using the Bader method [58,59]. This analysis is valuable since it allows us to describe the ionicity of the chemical bond by assessing the charge transfer between the atoms forming the bond [60].

In the structure of TiO_2_ and ZnTiO_3_, the net charge of Ti (+2.6e) was 1.4e less than its formal charge of +4e, while the oxygen (O) atom had a negative charge of −1.3e, which is 0.7e less than its formal charge of −2e. In the ZnTiO_3_ structure, the Zn atom had a positive charge of +1.3e, which is 0.7e less than its formal charge of +2e. These results align with previous findings [61,62]. For the CN^−^ species, the net charge of carbon © was +3.9e, which is 4.9e greater than its formal charge of −1e, while the net charge of nitrogen (N) was −3.9e, indicating a deviation of 3.9e from its formal charge. In the HCN species, the net charge of hydrogen (H) was +0.2e, which is 0.2e greater than its formal charge of 0.0e, while the net charges of carb©(C) and nitrogen (N) varied by 3.9e from their respective formal charges of 0.0e [63].

Based on the results in Table 7, it was observed that both the CN-TiO_2_ and CN-ZnTiO_3_ interactions exhibited an average decrease of 1.21e in the magnitude of the net charge of the CN^−^ species. Conversely, for both the HCN-TiO_2_ and HCN-ZnTiO_3_ interactions, there was an average decrease of 0.07e in the magnitude of the net charge of the HCN species. 

**Figure 11:**
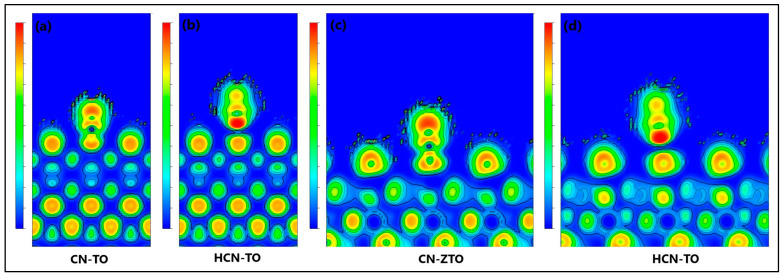
Representation of ELF with contour lines of (**a**) CN-TiO_2_, (**b**) HCN-TiO_2_, (**c**) CN-ZnTiO_3_, and (**d**) HCN-ZnTiO_3_.

Moreover, in this study, the electron localization function (ELF) was employed to gain a deeper understanding of the CN-TiO_2_, CN-ZnTiO_3_, HCN-TiO_2_, and HCN-ZnTiO_3_ interactions [64]. In ELF analysis, when the region of maximum density (RMD) is more symmetrically distributed around the nucleus, it signifies a more ionic or van der Waals interaction. Conversely, as the covalent character of a bond strengthens, RMD migration between centers becomes more pronounced until achieving a perfectly symmetric geometry in the ideal covalent scenario. In Figure 11, the ELF section for the CN-TiO_2_ and CN-ZnTiO_3_ interactions indicates an interaction between the nitrogen (N) atom of the CN^−^ species and the oxygen (O) atom on the surface of the semiconductors. The figure illustrates that the RMDs are positioned along the line connecting the nuclei and can be separated from the nuclei themselves by a path. Furthermore, the RMDs do not surround the nucleus [65]. 

### 2.4. Photodegradation Studies

**Figure 12:**
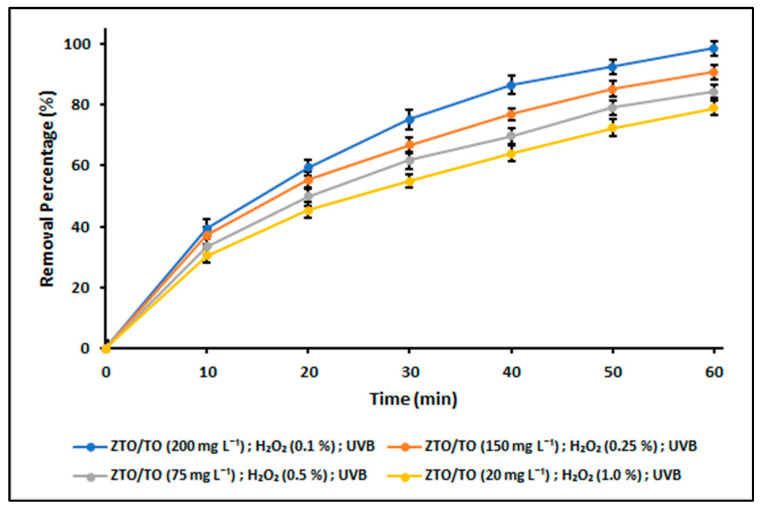
Cyanide photodegradation capacity as a function of catalyst concentration and H_2_O_2_ concentration. Cyanide concentration = 100 mg L^−1^, Solution pH = 10.0 ± 0.1, Temperature = 20 °C, n = 3.

The results obtained from the photodegradation experiments are presented in Figure 12. The photodegradation of cyanide species was evaluated using the ZnTiO_3_/TiO_2_/H_2_O_2_/UVB system in four different tests, varying the proportions of the hybrid photocatalyst ZnTiO_3_/TiO_2_ and the oxidant reagent H_2_O_2_. From the figure, it is evident that the highest percentage of cyanide photodegradation after 60 min of reaction under UVB light was achieved with the ZnTiO_3_/TiO_2_ (200 mg L^−1^)/H_2_O_2_ (0.10%) system. 

The Langmuir–Hinshelwood equation revealed a linear relationship between ln(C_0_/C_t_) and t, providing confirmation that the photocatalytic degradation reaction follows a pseudo-first-order kinetics. Table 8 presents the apparent rate constants (k_app_) for the photodegradation of cyanide species in the ZnTiO_3_/TiO_2_/H_2_O_2_/UVB system, determined for all the tests conducted. 

### 2.5. Optimization of Process Variables and Reuse of Nanoparticles

In order to optimize the cyanide species removal process, a total of 23 tests were carried out in which the following variables were combined: the ZnTiO_3_/TiO_2_ photocatalyst and the H_2_O_2_ oxidizing agent. The concentration of the hybrid photocatalyst was in the range of 20 to 200 mg L^−1^ while the concentration of the oxidizing agent was in the range of 0.1 to 1.0%. The results of the experiments in the absence and presence of UVB light are described in Table 9.

**Figure 13:**
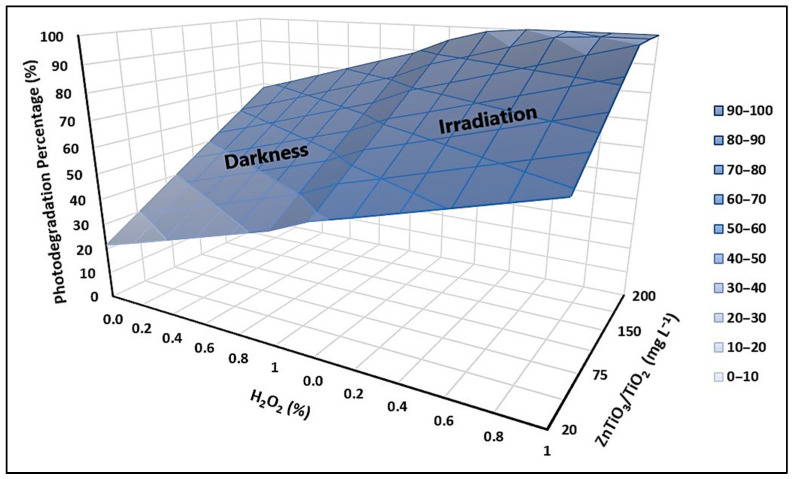
Response surface plot for optimization of the cyanide removal process.

To obtain a comprehensive understanding of the optimal composition of the reaction system, a response surface methodology (RSM) was used [66]. Figure 13 provides a visual representation of the relationship between UVB light, ZnTiO_3_/TiO_2_ concentration, and H_2_O_2_ concentration in achieving efficient cyanide removal. The graph clearly indicates that the presence of UVB light significantly enhances cyanide removal. Furthermore, it demonstrates that to attain maximum efficiency (100%) while minimizing H_2_O_2_ usage (0.4%), a ZnTiO_3_/TiO_2_ concentration of 200 mg L^−1^ is necessary.

**Figure 14:**
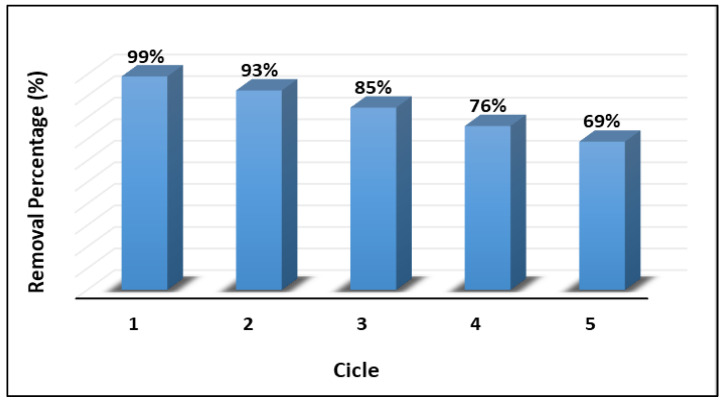
Cyanide removal efficiency of the ZnTiO_3_/TiO_2_/H_2_O_2_/UVB system for five cycles (Cyanide concentration = 100 mg L^−1^, Solution pH = 10.0 ± 0.1, Temperature = 20 °C, n = 3).

On the other hand, given that the stability and reusability of materials utilized in adsorbent and photocatalytic applications are decisive factors for their potential large-scale implementation, this study conducted a series of reuse experiments comprising five consecutive cycles of cyanide removal. Figure 14 illustrates the cyanide removal efficiency of the ZnTiO_3_/TiO_2_/H_2_O_2_/UVB system following 60 min of reaction, spanning these five cycles. The composition of the reaction system was as follows: ZnTiO_3_/TiO_2_ = 200 mg L^−1^, H_2_O_2_ = 0.1%, and UVB light irradiation.

### 2.6. Toxicity Studies

**Figure 15:**
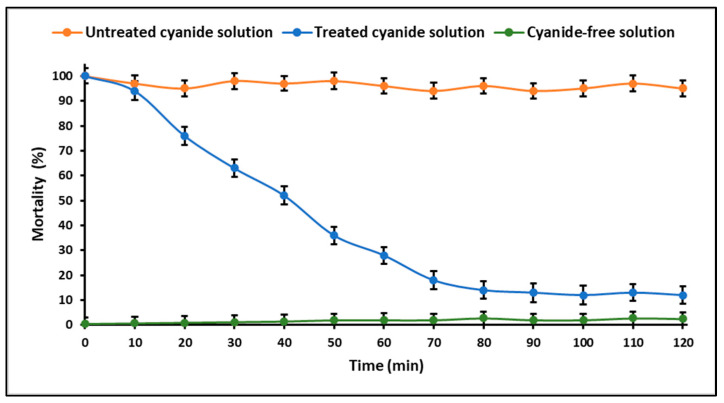
Acute toxicity profile in nauplii of the brine shrimp of the cyanide solutions treated by the system: ZnTiO_3_/TiO_2_ (200 mg g^−1^) + H_2_O_2_ (0.1%) + UVB light. (Cyanide concentration = 100 mg L^−1^, Solution pH = 9.0 ± 0.1, Temperature = 20 °C, n = 3).

As depicted in Figure 15, the detoxification effectiveness of the ZnTiO_3_/TiO_2_/UVB system was assessed using the brine shrimp toxicity test. In the absence of treatment, nearly all nauplii experienced near 100% mortality. However, as the treatment duration increased, a notable reduction in mortality became apparent, with mortality levels reaching a maximum of approximately 10% after 90 min of treatment. In contrast, the cyanide-free solution exhibited an insignificant percentage of nauplii mortality.

*Principio del formulario*

*Final del formulario*

## 3. Discussion

### 3.1. Characterization of the Nanoparticles

#### 3.1.1. XRD Analysis

XRD analysis provides information on the structural characteristics and dimensions of the synthesized ZnTiO_3_/TiO_2_ nanoparticles. The phases and sizes of the crystals observed are important since they directly influence the photocatalytic properties of the semiconductor. The diffraction pattern shown in Figure 1 confirms the presence of the ZnTiO_3_ and TiO_2_ phases in the catalyst, so based on previous results, it is inferred that the composite material could exhibit improved photocatalytic activity compared to the individual components. Furthermore, the relatively small crystallite sizes suggest the presence of a high surface area, which would facilitate a greater interaction between the photocatalyst and the target contaminant in aqueous solutions, potentially leading to improved photocatalytic performance.

#### 3.1.2. SSA and pH_PZC_ Analysis

The specific surface area (SSA) of the nanoparticles is an important parameter since, as mentioned in the previous section, it is directly related to the surface area available for interactions with the target contaminant. A relatively large SSA suggests a high degree of surface exposure, which may facilitate greater adsorption and catalytic activity. The extensive surface area provides ample active sites for the binding of contaminants and the initiation of photocatalytic reactions. Therefore, the observed SSA of ZnTiO_3_/TiO_2_ nanoparticles is advantageous for its potential application in the removal of contaminants from aqueous solutions.

On the other hand, the identification of the point of zero charge (pH_PZC_) of nanoparticles is important as it provides information about their surface chemistry and charging behavior. In particular, the literature suggests that the presence of oxygen functional groups in adsorbent materials plays an essential role in determining their surface properties and basic characteristics [17]. In the case of ZnTiO_3_/TiO_2_ nanoparticles, the observed pH_PZC_ of 7.0 implies a slight predominance of basic functional groups on their surface. This surface chemistry could influence the adsorption and interaction of various molecules, particularly those with acidic or basic properties, in aqueous solutions. It is important to mention that Figure 2 shows that there is a sudden change in ΔpH at an initial pH of 10. As the pH increases above 7, the concentration of hydroxide ions (OH^–^) begins to increase sharply, resulting in a more alkaline medium. In addition to hydroxide ions, other ions may also be present in the solution depending on the chemical components used in the experiment. In fact, since in this study, NaOH was added to the solution to adjust the pH, sodium ions (Na^+^) would also be present. On the other hand, chloride ions (Cl^–^) would also be present from sodium chloride (NaCl), which is the salt used to prepare the solutions. Although sodium chloride does not have a direct effect on pH, it can influence the charge balance on the surface of the material and therefore the ΔpH, depending on the concentration used. In this way, it is suggested that the presence of the aforementioned chemical species in the reaction medium could alter the surface charge of ZnTiO_3_/TiO_2_, which would explain the abrupt change in ΔpH at an initial pH of 10.

The combination of a significant SSA and a pH_PZC_ of 7.0 suggests that ZnTiO_3_/TiO_2_ nanoparticles could be well suited for applications involving the removal of a wide range of contaminants from aqueous solutions. The presence of basic surface groups suggests potential interactions with acidic contaminants, while the large SSA provides an extensive surface area for the binding and subsequent degradation of target compounds during photocatalysis. These surface properties contribute to the overall effectiveness and versatility of ZnTiO_3_/TiO_2_ nanoparticles in environmental remediation applications.

#### 3.1.3. SEM and EDS Analysis

The SEM images in Figure 3a,b clearly show the morphology of TiO_2_ and ZnTiO_3_/TiO_2_ nanoparticles, respectively. Both materials have a nearly spherical shape and are densely packed. However, significant differences in the shape and tendency to form clusters were observed between the two semiconductors. TiO_2_ nanoparticles exhibited a more irregular shape compared to ZnTiO_3_/TiO_2_ nanoparticles. Furthermore, ZnTiO_3_/TiO_2_ nanoparticles tend to form clusters. Image analysis using ImageJ2 software version 1.54g allowed for determining the average size and standard deviation of the nanoparticles of each material. The results revealed that the size of TiO_2_ nanoparticles had an average value of 37.6 nm with a standard deviation of 7.2 nm. On the other hand, ZnTiO_3_/TiO_2_ nanoparticles showed an average size of 27.4 nm, with a standard deviation of 4.9 nm. The small size and nearly spherical shape of the nanoparticles contribute to their high specific surface area, as discussed above, which can be advantageous for various applications, including adsorption and photocatalysis.

When considering standard deviations, it is observed that both TiO_2_ and ZnTiO_3_/TiO_2_ nanoparticles exhibit some variability in particle size. However, the standard deviation of 7.2 nm in TiO_2_ nanoparticles indicates a slightly larger dispersion compared to ZnTiO_3_/TiO_2_ nanoparticles, which have a standard deviation of 4.9 nm. These data indicate that TiO_2_ nanoparticles have a broader size distribution compared to ZnTiO_3_/TiO_2_ nanoparticles. It is important to note that a larger standard deviation implies a wider spread of particle sizes around the average value. The difference in size distributions between ZnTiO_3_/TiO_2_ and TiO_2_ nanoparticles can have important implications for their properties and applications. Nanoparticles with a narrower and more homogeneous size distribution may be preferable in certain cases where uniformity and consistency in performance are sought. On the other hand, a broader distribution may be acceptable or even beneficial in applications that require specific properties related to size variability.

Regarding the specific elemental composition of the nanoparticles, shown in Figure 3c,d, both TiO_2_ and ZnTiO_3_/TiO_2_ have a relatively high proportion of titanium and oxygen, as expected from titanium oxide-based materials. In contrast, the detection of carbon in both samples is due to the use of sample holders based on this element. In fact, given the particle size of the dust samples, it is possible that during the test there were uncoated spaces, which provided the signal from the sample holder, that is, from the carbon. Finally, from the results shown in Table 2, it was determined that the ratio of zinc titanate to titanium oxide in the ZnTiO_3_/TiO_2_ nanoparticles is approximately 1:10. These findings support the results of the XRD analysis regarding the composition of the hybrid semiconductor and suggest potential applications in several fields, including environmental remediation and catalysis.

### 3.2. Optimization of Cyanide Removal Process

In this study, the cyanide removal capacity (q_e_) was first evaluated for different combinations of ZnTiO_3_/TiO_2_ and H_2_O_2_ concentrations. When analyzing Figure 4 and considering an initial cyanide concentration of 100 mg L^−1^, it is observed that both the ZnTiO_3_/TiO_2_ concentration and the H_2_O_2_ concentration influence the removal capacity of the system. In fact, it can be observed that a lower concentration of ZnTiO_3_/TiO_2_ results in a lower cyanide removal capacity. This suggests that the availability of adsorption sites on the surface of the photocatalyst plays an important role in capturing the cyanide present. As the concentration of ZnTiO_3_/TiO_2_ decreases, the number of active sites available for cyanide adsorption is reduced. Therefore, it is crucial to optimize the concentration of ZnTiO_3_/TiO_2_ to maximize the adsorption capacity. Likewise, in Figure 4, it is observed that as the H_2_O_2_ concentration decreases, the removal capacity also decreases. This implies that the oxidation and decomposition of cyanide species are affected by the amount of H_2_O_2_ present in the system. H_2_O_2_ plays an important role in the oxidation of cyanides, converting them into less toxic compounds. At a lower concentration of H_2_O_2_, the oxidation efficiency is reduced, which decreases the removal capacity of the system. However, it is important to note that the use of large quantities of H_2_O_2_ may be impractical on a large scale due to economic and safety considerations. Therefore, it is necessary to find a balance between adsorption efficiency and the amount of H_2_O_2_ used, taking into account the practical aspects of large-scale treatment.

It is important to mention that the removal capacity presented in Figure 4 for the ZnTiO_3_/TiO_2_/H_2_O_2_ system is generally considerable. However, it is essential to note that these results are based on the experimental conditions used in this study. Therefore, additional research considering a variety of conditions and scales is necessary to achieve a more complete and accurate understanding of the performance of cyanide treatment with the ZnTiO_3_/TiO_2_ and H_2_O_2_ system in different situations.

On the other hand, the results of the analysis of variance (ANOVA) described in Table 2 confirm the importance of the ZnTiO_3_/TiO_2_ concentration and the H_2_O_2_ concentration on the cyanide removal capacity. The variations in the values of q_e_ between the different compositions of the system are not random but are due to the deliberate manipulation of these two factors. Consequently, it is essential to optimize the concentrations of the semiconductor and the oxidizing agent to achieve the highest cyanide removal capacity. From the results obtained, a specific composition of 200 mg L^−1^ of ZnTiO_3_/TiO_2_ and 0.1% of H_2_O_2_ was chosen to continue with the cyanide removal experiments, in order to achieve a balance between effective cyanide removal and practicality.

### 3.3. Adsorption Studies

#### 3.3.1. Effect of pH on Cyanide Adsorption

As seen in Figure 5, the pH of the solution plays a crucial role in influencing the adsorption of ions on the surface of ZnTiO_3_/TiO_2_ nanoparticles. The pH affects both the surface charge of the adsorbent material and the ionization state of the ions in the solution. The adsorption mechanisms at play in the case of cyanide species on nanoparticle surfaces depend on the pH-dependent distribution of cyanide species in the solution [17].

Inorganic cyanides, such as KCN and NaCN, have the characteristics of weak acids and undergo hydrolysis reactions. At pH levels below 9.4, hydrocyanic acid (HCN) becomes the dominant species in the aqueous solution. In contrast, at pH levels above 9.4, HCN begins to dissociate into H^+^ and CN^−^ ions [67]. It is important to mention that several investigations have been carried out on the adsorption of cyanide at pH values of approximately 7.0 (where pH is lower than the pKa value), suggesting that the strong adsorption of HCN species would be driven by interactions with the existing Lewis acid sites on the adsorbent surface [68,69]. CN^−^ ions, on the other hand, exhibit nucleophilic properties and show pronounced electrostatic attraction in solutions with a pH greater than 9.0. This observation has led several researchers to indicate a higher cyanide adsorption capacity within the pH range of 9 to 11 [56]. 

The results indicate that the combined adsorption capacity for total cyanide (HCN + CN^−^) by ZnTiO_3_/TiO_2_ nanoparticles remained relatively stable within the pH range of 9 to 11, even though the point of zero charge (pH_PZC_) of the nanoparticles was 7.0. This suggests that the adsorption of cyanide species on the surface of nanoparticles can be influenced by multiple mechanisms, including interactions with Lewis acid sites and electrostatic forces.

#### 3.3.2. Maximum Cyanide Adsorption Capacity

Langmuir, Freundlich, and Temkin isotherms were used to assess their suitability as equilibrium models to describe the adsorption of cyanide species on ZnTiO_3_/TiO_2_ nanoparticles. The Langmuir model, which assumes uniform surface adsorption energies and no lateral transmigration of the adsorbate on the surface, was found to fit the cyanide adsorption data remarkably well. The Freundlich isotherm, which is exponential in nature, suggests an increase in the adsorbate concentration at the surface of the adsorbent as the adsorbate concentration increases. The Temkin isotherm postulates that the heat of adsorption for all molecules in the adsorbed layer decreases linearly with coverage due to interactions between the adsorbent and adsorbate [70].

The experimental results demonstrate that the Langmuir model fits the cyanide adsorption data remarkably well across a range of temperatures. The separation factor RL, a critical parameter in the Langmuir model, supports the favorable nature of cyanide adsorption on ZnTiO_3_/TiO_2_ nanoparticles. This finding suggests that the adsorption process is not only favorable but also very efficient. The excellent agreement between the Langmuir model and the experimental data suggests that the adsorption of cyanide species on ZnTiO_3_/TiO_2_ nanoparticles follows a monolayer adsorption mechanism with uniform adsorption energies. This insight is essential to understand and optimize the adsorption process in environmental and wastewater treatment applications.

#### 3.3.3. Adsorption Thermodynamics

Thermodynamic parameters, including the Gibbs free energy change (∆G°), enthalpy change (∆H°), and surface entropy change (∆S°), were determined to shed light on the spontaneity and feasibility of the adsorption process under various temperature conditions. The Gibbs free energy change (∆G°), which serves as a key indicator of the spontaneity of a process, showed negative values, supporting a spontaneous and highly favorable adsorption process. The negative values of ∆G° at all tested temperatures suggest that the adsorption of cyanide species on ZnTiO_3_/TiO_2_ nanoparticles is thermodynamically favorable and spontaneous. Furthermore, the progressively more negative ∆G° values with increasing temperature further emphasize the spontaneous nature of the adsorption process. On the other hand, the enthalpy change (∆H°) indicates that the adsorption process is endothermic. Positive ∆H° values suggest that the adsorption of cyanide species onto the nanoparticles releases heat into the surroundings, indicating an endothermic reaction. Finally, the surface entropy change (∆S°) offers insights into the change in randomness at the solution–solid interface during the adsorption process. A positive ∆S° value indicates an increase in randomness. The provided value of ∆S° suggests a major increase in entropy at the interface, which aligns with the adsorption process.

#### 3.3.4. Kinetic of Cyanide Adsorption

Figure 8 illustrates the behavior of three kinetic models, namely pseudo-first-order, pseudo-second-order, and Elovich models, in relation to cyanide adsorption. All three models show an initial phase of rapid adsorption followed by a plateau phase. However, the crucial distinction lies in their ability to accurately represent experimental data. Table 4 provides an overview of the model performance based on its correlation coefficients (R^2^).

Among the three models, the pseudo-second-order model stands out with the highest correlation coefficient (R^2^ = 0.99), indicating that it provides the best fit for the experimental data. This observation suggests that the adsorption of cyanide species on ZnTiO_3_/TiO_2_ nanoparticles is likely governed by a chemisorption process, which is consistent with the existing literature [20]. The pseudo-second-order model is based on the assumption that the rate-limiting step of adsorption involves the sharing or exchange of electrons between the adsorbent surface and the adsorbate. The kinetic parameters obtained for this model, including the maximum adsorption capacity (q_max_) and the rate constant (k_2_), are valuable for understanding and predicting the kinetics of the adsorption process.

While the Elovich model also provides a reasonable fit to the data (R^2^ = 0.95), it appears to be slightly less accurate than the pseudo-second-order model. The Elovich model describes adsorption as a multilayer process involving interactions between the adsorbent surface and adsorbate molecules. Despite its slightly lower precision, the Elovich model can still provide valuable insights into adsorption kinetics. In addition to the kinetic models, the intraparticle diffusion model was employed to obtain more insights into the rate-limiting step of the adsorption process. Figure 9 demonstrates the changes in the cyanide adsorption curve over time, and Table 6 presents the estimated kinetic parameters of this model. The relatively low value of R^2^ (0.84) suggests that intraparticle diffusion alone does not entirely govern the adsorption process; instead, multiple kinetic steps may be involved. In fact, Figure 9 indicates that two steps occurred in the adsorption process. The first adsorption stage had the largest slope and was attributed to external surface adsorption or instantaneous adsorption, while the second adsorption stage showed a moderate slope compared to the first, indicating a gradual adsorption stage. Consequently, the rate-limiting step was diffusion within the particles [71,72].

#### 3.3.5. DFT Study of the Cyanide Adsorption onto ZnTiO_3_/TiO_2_ Nanoparticles

The objective of the computational analysis using the DFT method was to obtain information on the adsorption mechanisms and chemical interactions between the cyanide species CN^−^ and HCN and the surfaces (101) of the semiconductor structures of TiO_2_ and ZnTiO_3_. Bader’s charge analysis revealed a net transfer of charge between the atoms involved in the chemical bonds. Specifically, for CN-TiO_2_ and CN-ZnTiO_3_ interactions, there was an average decrease of 1.21e in the magnitude of the net charge of CN^−^ species. In contrast, for both HCN-TiO_2_ and HCN-ZnTiO_3_ interactions, there was an average decrease of 0.07e in the magnitude of the net charge of the HCN species. According to the literature, a non-zero Bader charge transfer signifies a bond ionicity equal to zero. Similarly, a very small charge transfer, approximately 0.05e, between bonded atoms indicates weak bond ionicity. In contrast, a substantial charge transfer (e.g., 1.5e) indicates significant bond ionicity [73]. Therefore, in line with previous research, the results from Bader’s analysis suggest that a charge transfer occurs between the cyanide species and the semiconductor surfaces, leading to changes in the charge distribution around these atoms. The magnitude of these charge transfers indicates a moderate ionic character in the CN-TiO_2_ and CN-ZnTiO_3_ bonds, while the HCN-TiO_2_ and HCN-ZnTiO_3_ bonds appear to have a more covalent character [59,60].

Furthermore, electron localization function (ELF) analysis provided additional insights into the nature of the interactions. The ELF sections for CN-TiO_2_ and CN-ZnTiO_3_ interactions indicated connections between the nitrogen (N) atom of the CN^−^ species and the oxygen (O) atom on the surface of the semiconductors. The distribution of the region of maximum density (RMD) along the line connecting the nuclei and its separation from the nuclei themselves by a path suggests the generation of polar covalence in these bonds. This observation is consistent with a covalent character in the CN-TiO_2_ and CN-ZnTiO_3_ interactions. These findings collectively suggest that the adsorption of cyanide species on the semiconductor surfaces involves chemical interactions characterized by weak to moderate ionic and covalent bonds. Understanding these interactions is important for the design and optimization of adsorption processes involving cyanide removal using TiO_2_ and ZnTiO_3_ semiconductor materials. 

However, it should be noted that in the context of materials selectivity for cyanide removal, the lack of selectivity can be a major challenge in the application of these materials in environmental pollution remediation. In this regard, experimental and theoretical studies play complementary roles in understanding and addressing material selectivity in cyanide adsorption. Experimental studies can evaluate the adsorption capacity of different materials in the presence of various cyanide species, as well as the influence of other components present in the aqueous solution. On the other hand, theoretical studies, such as molecular simulation and energy calculations, can provide valuable information on the molecular interactions between the material and cyanide. These theoretical approaches can help understand the mechanisms of cyanide adsorption and displacement, as well as predict the relative selectivity of different materials based on their structural and chemical properties. Consequently, the evidence from this study suggests that the combination of experimental and theoretical studies could allow for a better understanding of adsorption mechanisms and the development of more effective strategies for the selective removal of cyanide, thus contributing to the mitigation of environmental pollution.

### 3.4. Photodegradation Studies

The photodegradation of cyanide species in aqueous solutions has received significant attention in previous research due to the promising capabilities of titanium-based semiconductor materials as photocatalysts under ultraviolet (UV) radiation. In this study, we extend the exploration of photocatalytic degradation of cyanide to a hybrid semiconductor, ZnTiO_3_/TiO_2_, and investigate its performance under UVB radiation, taking into account the influence of hydrogen peroxide (H_2_O_2_) in the reaction system.

The results of our photodegradation experiments, as shown in Figure 12, highlight the effectiveness of the ZnTiO_3_/TiO_2_ photocatalyst and the oxidizing agent H_2_O_2_ in removing cyanide species from aqueous solutions. We performed four different tests, varying the proportions of ZnTiO_3_/TiO_2_ and H_2_O_2_ in the reaction system. The ZnTiO_3_/TiO_2_ (200 mg L^−1^)/H_2_O_2_ (0.10%) system exhibited the highest percentage (99%) of cyanide photodegradation after 60 min of exposure to UVB light. This finding is significant as it demonstrates the exceptional potential of the ZnTiO_3_/TiO_2_ hybrid semiconductor to achieve complete photodegradation of cyanide species while reducing the consumption of the oxidizing agent H_2_O_2_.

Furthermore, we evaluated the kinetics of the photodegradation reaction using the Langmuir–Hinshelwood equation. Table 8 presents the apparent rate constants (k_app_) for the photodegradation of cyanide species in the various test systems. The ZnTiO_3_/TiO_2_ (200 mg L^−1^)/H_2_O_2_ (0.10%) system exhibited the highest k_app_ value, further supporting the superior performance of this configuration in facilitating the rapid degradation of cyanide species.

The heterojunction between ZnTiO_3_ and TiO_2_ serves as a strategic interface that enables a cascade of advantageous effects in the photocatalytic system. First, this interface greatly improves the separation of photogenerated charge carriers (electrons and holes). Photogenerated electrons (e^−^) and holes (h^+^) tend to recombine rapidly in traditional photocatalytic materials, limiting their effectiveness. However, in the ZnTiO_3_/TiO_2_ heterojunction, the interface acts as a charge sink, effectively trapping the electrons and preventing their immediate recombination with holes. This prolongs the lifetime of photogenerated charge carriers, ensuring their availability to participate in photocatalytic reactions. Secondly, the heterojunction enhances the interfacial charge transfer to adsorbed substrates. When contaminants such as cyanide species are adsorbed on the surface of the photocatalyst, the heterojunction facilitates efficient charge transfer to these adsorbates. This rapid charge transfer initiates redox reactions on the surface of the contaminants, leading to their degradation.

Furthermore, the introduction of hydrogen peroxide (H_2_O_2_) into the reaction system plays a critical role in boosting the generation of reactive oxygen species (ROS). These ROS, including hydroxyl radicals (^•^OH) and superoxide radicals (^•^O_2_^−^), are highly reactive and possess strong oxidative capabilities. In the presence of the ZnTiO_3_/TiO_2_ heterojunction and UVB radiation, H_2_O_2_ undergoes photocatalytic activation, resulting in the production of ROS. These ROS are particularly fundamental in two essential aspects of the process. First, they react directly with cyanide species, initiating oxidation reactions that ultimately lead to the degradation of cyanide compounds. This direct oxidation is a fundamental step in the photocatalytic degradation mechanism. Second, ROS also contributes to the regeneration of photogenerated charge carriers (e^−^ and h^+^). Hydroxyl radicals (^•^OH) can trap photogenerated holes (h^+^) and convert them into hydroxide ions (OH^−^), while superoxide radicals (^•^O_2_^−^) can react with photogenerated electrons (e^−^) to produce superoxide ions (^•^O_2_^−^). These regenerated charge carriers can continue to participate in the degradation of cyanide species, thus enhancing the overall photocatalytic efficiency.

### 3.5. Optimization of Process Variables and Reuse of Nanoparticles

The effectiveness of the ZnTiO_3_/TiO_2_ hybrid photocatalyst in cyanide removal was further investigated by examining the impact of varying its concentration in the presence of H_2_O_2_ under UVB light. These experiments aimed to understand the relationship between catalyst concentration, H_2_O_2_ concentration, and cyanide removal efficiency. The utilization of H_2_O_2_ is an important option for CN oxidation of free and weakly complexed metals as follows [10]:

(20) $${CN}^{-}+H_{2}O_{2}\to {OCN}^{-}+H_{2}O$$

(21) $$M{\left(CN\right)}_{4}^{2-}+4H_{2}O_{2}+2{OH}^{-}\to {4OCN}^{-}+4H_{2}O+{M\left(OH\right)}_{2}(solid)$$

The excess H_2_O_2_ used for the oxidation of CN can be decomposed to produce oxygen and water; however, the use of large quantities of this oxidizing agent in large-scale processes is impractical [12]. 

Additionally, cyanate formed during the reactions can be hydrolyzed to bicarbonate and ammonia. Generally, almost 10–20% of CN is hydrolyzed to ammonia in the treatment process as follows [10]:

(22) $${OCN}^{-}+H^{+}+{2H}_{2}O\to {HCO}_{3}^{-}+{NH}_{4}^{+}$$

The results, summarized in Table 9, provide important insights into how to optimize the composition of the reaction system for efficient cyanide removal. In the absence of light, increasing the concentration of the ZnTiO_3_/TiO_2_ hybrid photocatalyst by 10 times, from 20 to 200 mg L^−1^, while keeping the H_2_O_2_ concentration constant, led to a notable improvement of approximately 37% in the cyanide removal efficiency. In contrast, when the concentration of H_2_O_2_ was increased by 10 times, from 0.1 to 1.0%, while maintaining a constant amount of ZnTiO_3_/TiO_2_, the reaction system exhibited a 10% increase in cyanide removal efficiency. These results suggest that variations in catalyst concentration have a more significant impact on the rate of cyanide removal compared to changes in H_2_O_2_ concentration.

For the photocatalytic degradation experiments performed under UVB light, different combinations of the ZnTiO_3_/TiO_2_ concentration and H_2_O_2_ concentration were explored to understand their effects on cyanide removal. In particular, when UVB light was present, the highest cyanide removal efficiency of 99.74% was achieved with a ZnTiO_3_/TiO_2_ concentration of 200 mg L^−1^ and an H_2_O_2_ concentration of 0.1%. This result underscores the crucial role of UVB light in enhancing the photocatalytic degradation of cyanide.

Regarding the findings shown in the response surface plot from Figure 13, these have practical implications for the design of cyanide removal processes using the ZnTiO_3_/TiO_2_ hybrid photocatalyst. By carefully optimizing the composition of the reaction system, it is possible to achieve efficient and environmentally friendly removal of cyanide from aqueous solutions. 

On the other hand, the long-term stability and reusability of materials used in adsorbent and photocatalytic applications are critical factors to consider for their practical and large-scale implementation. Therefore, a series of reuse experiments were conducted to assess the performance and robustness of the ZnTiO_3_/TiO_2_/H_2_O_2_/UVB system in cyanide removal. These experiments involved five consecutive cycles of cyanide removal. Figure 14 provides a visual representation of the cyanide removal efficiency achieved by the ZnTiO_3_/TiO_2_/H_2_O_2_/UVB system after 60 min of reaction over these five cycles. The results indicate that the hybrid photocatalyst retains its catalytic activity even after multiple cycles of use, with only a slight decrease observed over time. The cyanide removal efficiency remained remarkably satisfactory across the five consecutive cycles. The ability of the ZnTiO_3_/TiO_2_ hybrid photocatalyst to maintain its efficacy over repeated applications is a promising characteristic for real-world applications. It implies that the material can be reused without a significant loss of catalytic performance, making it a cost-effective and environmentally friendly option for treating cyanide-contaminated water sources. Furthermore, the preservation of catalytic activity over multiple cycles is indicative of the material’s stability and durability, further supporting its potential for practical use.

### 3.6. Toxicity Studies

The detoxification efficiency of the ZnTiO_3_/TiO_2_/H_2_O_2_/UVB system was assessed by a brine shrimp toxicity test. Figure 15 illustrates the results of this toxicity test, highlighting the significant impact of the treatment on the survival of brine shrimp nauplii. In the absence of any treatment, the mortality rate among the brine shrimp nauplii approached 100%, indicating the serious toxicity of the cyanide-containing solution. However, as the duration of treatment with the ZnTiO_3_/TiO_2_/H_2_O_2_/UVB system increased, a clear trend of reduced mortality emerged. In particular, after 90 min of treatment, the mortality rate of the brine shrimp nauplii reached a maximum of approximately 10%. This significant reduction in mortality is a testament to the detoxification capability of the ZnTiO_3_/TiO_2_/UVB system in effectively degrading cyanide species, rendering the solution less toxic to aquatic life. Furthermore, it is essential to highlight the comparison with the cyanide-free solution, which exhibited an insignificant percentage of nauplii mortality. This serves as a control group, confirming that the mortality reduction observed in the treated solution is attributed to the treatment process rather than any inherent toxicity of the experimental setup.

The brine shrimp toxicity test results demonstrate the potential of the ZnTiO_3_/TiO_2_/UVB system to detoxify cyanide-contaminated water, rendering it less harmful to aquatic organisms. This is a crucial consideration for environmental applications, as it suggests that the system can contribute to the remediation of cyanide contamination in natural water bodies and ultimately safeguard aquatic ecosystems.

## 4. Materials and Methods

### 4.1. Materials

In the course of this study, the subsequent analytical-grade substances were employed in their as-received state, devoid of any additional purification: Titanium (IV) isopropoxide (Ti(OC_3_H_7_)_4_, Sigma Aldrich, St. Louis, MO, USA, 98.0%), Isopropyl alcohol (C_3_H_8_O, Sigma Aldrich, St. Louis, MO, USA, ≥99.5%), Zinc acetate dihydrate (Zn(CH_3_COO)_2_∙2H_2_O, ACS, St. Louis, MO, USA, ≥98.0%), Acetic acid (CH_3_COOH, Sigma Aldrich, St. Louis, MO, USA, 99.8%), Hydrogen peroxide solution (H_2_O_2_, Sigma Aldrich, St. Louis, MO, USA, 30.0% in H_2_O), Hydrochloric acid (HCl, Sigma Aldrich, St. Louis, MO, USA, 37.0%), Sodium hydroxide (NaOH, Sigma Aldrich, St. Louis, MO, USA, ≥85.0%), Potassium cyanide (KCN, Sigma Aldrich, St. Louis, MO, USA, ≥97.0%), Picric acid ((O_2_N)_3_C_6_H_2_OH, Sigma Aldrich, St. Louis, MO, USA, ≥99.0%), Sodium carbonate (Na_2_CO_3_, Sigma Aldrich, St. Louis, MO, USA, ≥99.0%), Sodium chloride (NaCl, Sigma Aldrich, St. Louis, MO, USA, ≥99.0%), Potassium chloride (KCl, Sigma Aldrich, St. Louis, MO, USA, ≥99.0%), Magnesium chloride hexahydrate (MgCl_2_∙6H_2_O, Sigma Aldrich, St. Louis, MO, USA, ≥99.0%), Sodium sulfate (Na_2_SO_4_, Sigma Aldrich, St. Louis, MO, USA, ≥99.0%), and Calcium chloride dihydrate (CaCl_2_∙2H_2_O, Sigma Aldrich, St. Louis, MO, USA, ≥99.0%).

### 4.2. Synthesis of the ZnTiO_3_/TiO_2_ Nanoparticles

**Figure 16:**
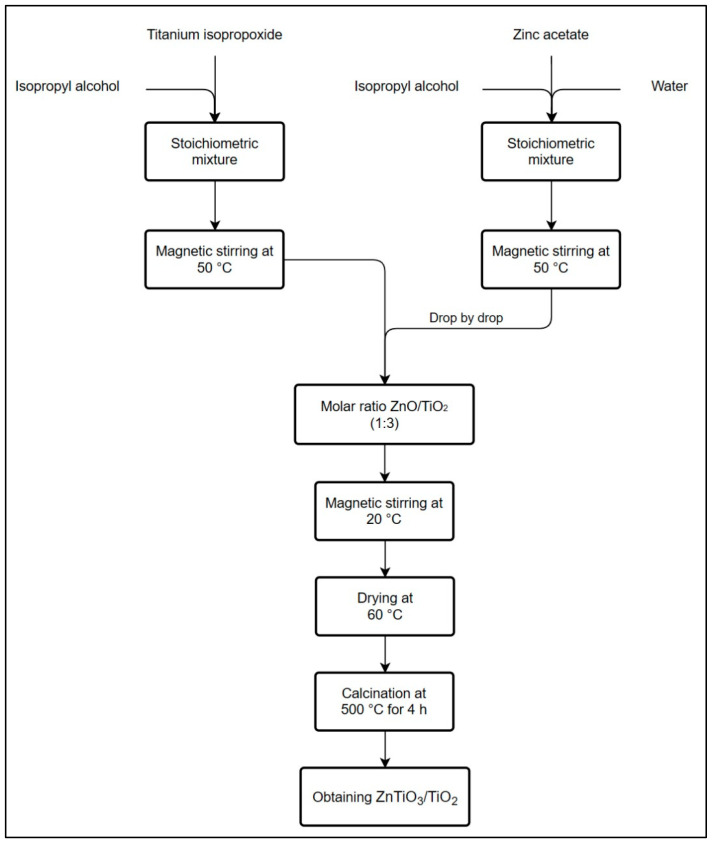
Schematic representation of the methodology for the synthesis of ZnTiO_3_/TiO_2_ nanoparticles.

The synthesis of ZnTiO_3_/TiO_2_ nanoparticles (referred to as ZTO/TO NPs) followed a sol–gel methodology as outlined in previous publications [74]. To prepare the ZTO/TO NPs, an alcoholic solution (designated as Solution 1) comprising titanium (IV) isopropoxide (TIPP) and isopropyl alcohol (iPrOH) was formulated with a TIPP/iPrOH ratio of 70 *v*/*v*%. Concurrently, an aqueous solution (referred to as Solution 2) was prepared, consisting of zinc acetate dihydrate (Zn(acet)), water (H_2_O), and isopropyl alcohol (iPrOH). In the preparation of Solution 2, a molar ratio of 3:1 for TiO_2_/ZnO was employed, along with a volumetric ratio of 1:1 for iPrOH/H_2_O, such that the quantity of H_2_O used could facilitate the hydrolysis of TIPP within the iPrOH medium. Subsequently, Solution 2 was incrementally added to Solution 1 under continuous agitation at room temperature. Following the formation of a white precipitate, the reaction mixture was stirred for a duration of 60 min at room temperature. The resultant precipitate was subjected to drying at 60 °C for 24 h, followed by calcination at 500 °C for a period of 4 h. Ultimately, the resulting solid materials were allowed to cool to room temperature. The scheme in Figure 16 provides an overview of the methodology for the synthesis of ZnTiO_3_/TiO_2_ nanoparticles.

### 4.3. Characterization of the Nanoparticles

The characterization of the ZnTiO_3_/TiO_2_ nanoparticles (ZTO/TO NPs) included a comprehensive suite of analytical techniques. X-ray diffraction (XRD) measurements were executed employing a Bruker-AXS D8-Discover diffractometer (Bruker AXS, Karlsruhe, Germany) equipped with Cu Kα radiation (λ = 1.5406 Å). Data acquisition spanned an angular range from 5 to 90° in terms of 2θ. To identify the crystalline phases present, the Crystallography Open Database (COD database, version 2021) was employed.

The specific surface area (SSA) of the ZTO/TO NPs was determined using a ChemiSorb 2720 instrument (Micromeritics, Norcross, GA, USA) through liquid nitrogen physisorption analysis performed at −196 °C. This analysis involved the use of a gas mixture comprising 30% nitrogen (N_2_) diluted in helium (He). The SSA was calculated utilizing the Brunauer–Emmet–Teller (BET) equation, employing the Chemisoft TPx system (version 1.03; data analysis software; Micromeritics, Norcross, GA, USA, 2011) via the single-point method.

Micrographs and energy-dispersive X-ray (EDX) spectra were acquired using a JEOL JSM 6400 scanning electron microscope (SEM) (JEOL, Peabody, MA, USA). The photoactivity of the samples was assessed at a wavelength of 310 nm using the IPW-UV-610 Stainless Steel Inner Sterilizer Light (IPW Industries Inc., Santa Ana, CA, USA), while the quantification of residual cyanide in the solutions was conducted at a wavelength of 490 nm employing a Jenway 7350 spectrophotometer (Cole-Parmer, Staffordshire, UK).

Furthermore, the point of zero charge (PZC) for the ZTO/TO NPs at room temperature (20 ± 1.0 °C) was determined utilizing the pH drift method, wherein ΔpH = pH_f_ − pH_i_ = 0. This experimental procedure involved adding 0.1 g of the solid sample to a 50 mL tube containing 25 mL of a 0.1 M NaCl solution. The pH of these solutions was adjusted to values ranging from 6 to 13 using 0.1 M HCl or NaOH solutions, and these values were recorded as pH_i_. The tubes were agitated for 24 h at 250 rpm, and the final pH of the supernatant liquid in each tube, denoted as pH_f_, was measured. By plotting ΔpH (ΔpH = pH_f_ − pH_i_) against pH_i_, the PZC was determined. This process was replicated for the ZTO/TO NPs using 0.01 and 0.05 M NaCl solutions. All experiments were conducted in triplicate, and the average pHPZC value was reported [75].

Lastly, IBM SPSS software (version 25.0; statistical software for Windows; IBM Corp.; Armonk, NY, USA, 2017) was employed for the analysis of variance (ANOVA).

### 4.4. Adsorption Studies

#### 4.4.1. Experimental Methodology

The investigation involved a series of cyanide species adsorption experiments on ZnTiO_3_/TiO_2_ nanoparticles (ZTO/TO NPs) using aqueous KCN solutions. These experiments were designed to assess the influence of various factors, namely (a) the solution pH, (b) the initial adsorbate concentration, (c) the reaction system temperature, and (d) the contact time between adsorbate and adsorbent. The data obtained from these adsorption experiments were subjected to fitting with isotherm and kinetic models through the least-squares non-linear regression method [70].

The adsorption experiments involving cyanide species were conducted in a batch reactor while maintaining the pH of the solutions at 10 ± 0.1. This pH control was achieved by introducing 0.1 M solutions of hydrochloric acid or sodium hydroxide. Additionally, the experiments were carried out at room temperature (20 ± 1.0 °C), with a constant catalyst quantity of 0.2 g L^−1^. To investigate the maximum cyanide adsorption capacity, the concentration of a 500 mL KCN solution was varied within the range of 2.5 to 100 mg L^−1^. Moreover, the adsorption thermodynamics and kinetic behavior of cyanide adsorption were explored utilizing a 500 mL water sample containing 100 mg L^−1^ of KCN. 

The alkaline picrate analytical method was employed to quantify the total cyanide content in aqueous solutions [76,77,78]. In this method, an alkaline picrate solution (PAS) was prepared, consisting of 1 g of picric acid ((O_2_N)_3_C_6_H_2_OH) and 5 g of sodium carbonate (Na_2_CO_3_) dissolved in 200 mL of HPLC-grade water. Subsequently, 4 mL of this PAS was added to 1 mL of either the cyanide solution or a blank solution (comprising HPLC-grade water) in a test tube. This tube was then incubated for 5 min in a water bath maintained at 95 °C. Following incubation, the absorbance at 490 nm was measured using a UV-visible spectrophotometer. Concentration determination relied on a pre-established calibration curve (R^2^ = 0.9994) in accordance with the Lambert–Beer Law. Triplicate experiments were conducted, and the results were expressed as the average of three repetitions. The quantity of cyanide adsorbed onto the nanoparticles was estimated using Equation (2), as presented in Table 1 [79].

The total equilibrium cyanide adsorption was assessed based on the Langmuir [80], Freundlich [81], and Temkin [82] isotherm models, each expressed by Equations (3)–(5), respectively, as outlined in Table 1. Furthermore, the constants pertaining to heat adsorption (B) and the separation factor (R_L_), providing insights into adsorption characteristics, were calculated employing Equations (6) and (7), also detailed in Table 1 [80].

For thermodynamic investigations, the experimental data were fitted based on the parameters of thermodynamic laws, specifically Gibbs free energy (∆G^0^, kJ mol^−1^), enthalpy (∆H^0^, kJ mol^−1^), and entropy (∆S^0^, kJ mol^−1^ K^−1^). These parameters were determined conventionally using Equation (8) provided in Table 1 [83]. Additionally, the relationship between ∆G^0^, ∆H^0^, and ∆S^0^ was deduced through the well-established van’t Hoff equation, denoted as Equation (9) in Table 1. In this equation, k_C_ is a dimensionless parameter, calculated by multiplying k_L_ by the molecular weight of the adsorbate (M_w_, g mol^−1^), and then further adjusting for factors 1000 and 55.5, representing the number of moles of pure water present in a liter, as elucidated in Equation (10) in Table 1 [84].

The kinetics of adsorption were investigated utilizing pseudo-first-order, pseudo-second-order, Elovich, intraparticle diffusion, external-film diffusion, and internal-pore diffusion models [81,82], each characterized by the respective mathematical expressions (11)–(16), all detailed in Table 1.

Furthermore, after the adsorption test, the materials that had adsorbed cyanide were subjected to a leaching process in water at pH 4, 7, and 10 for 24 h at room temperature. During this period, constant agitation was carried out to simulate leaching conditions in an aqueous environment. After the leaching period, an analysis was performed to determine the presence of cyanide in the aqueous solution.

#### 4.4.2. Theoretical Methodology

The Density Functional Theory (DFT) study was conducted employing the Vienna Ab Initio Simulation Package (VASP) version 6.0, developed by VASP Software GmbH in Vienna, Austria [85,86]. For the modeling and visualization of molecular structures, the molecular modeling software BioVia Materials Studio, version 5.5, provided by BioVia in San Diego, CA, USA, was employed.

In this study, the ionic potential of both inner nuclei and electrons was represented using pseudopotentials, which followed the projector augmented wave (PAW) methodology [87]. All calculations were performed utilizing the Perdew–Burke–Ernzerhof (PBE) generalized gradient approximation (GGA) function to describe electronic exchange-correlation interactions [88]. The plane wave cutoff energy was set to 500 eV. The Kohn–Sham equations [89] were solved in a self-consistent manner, ensuring that the energy variation between computational cycles remained below 10^−5^ eV.

The molecular adsorption of cyanide species on the (101) surface of ZnTiO_3_ was simulated using the following optimized parameters: a hexagonal ZnTiO_3_ crystal structure with lattice dimensions of a = 5.15 Å, b = 5.15 Å, and c = 13.94 Å, with angles between the lattice vectors <90° × 90° × 120°> [62]. Semiconductor properties were assessed by sampling the first Brillouin zone with Monkhorst–Pack [90] k-point meshes of 3 × 2 × 1. Similarly, the molecular adsorption of cyanide species on the (101) surface of TiO_2_ in its anatase phase was modeled with the following optimized parameters: a tetragonal TiO_2_ crystal structure with lattice parameters a = 3.82 Å, b = 3.82 Å, and c = 9.70 Å, and angles between the lattice vectors <90° × 90° × 90°> [61]. Semiconductor properties were estimated using Monkhorst–Pack k-point meshes of 1 × 3 × 1. All calculations were conducted under non-spin-polarized conditions. To enhance the convergence of the total energy, the Gaussian smearing method was employed with a width (σ) of 0.10 eV. Atomic positions were relaxed until the respective forces were reduced to less than 0.001 eV/Å.

For the study of cyanide species adsorption, both the ZnTiO_3_ and TiO_2_ materials were cleaved at their stable (101) surfaces [91,92,93]. The ZnTiO_3_ (101) slab model was constructed as a p(2 × 3) supercell, containing 36 Zn atoms, 36 Ti atoms, and 108 O atoms. Conversely, the TiO_2_ (101) slab model was formulated as a p(3 × 3) supercell, comprising 168 Ti atoms and 336 O atoms. The surface energies (γ_s_) for the ZnTiO_3_ and TiO_2_ structures, with a vacuum distance of 20 Å, were calculated using Equation (17) as described in Table 1 [94,95]. Additionally, the adsorption energy (E_ads_) of cyanide species on the (101) surface of both ZnTiO_3_ and TiO_2_ oxides was determined using Equation (18) outlined in Table 1 [96,97].

To elucidate the chemical characteristics of the cyanide–semiconductor interaction, a population analysis was conducted using Bader’s method. This analysis serves as a valuable tool for assessing the ionicity of a bond by quantifying the charge transfer between the atoms participating in the chemical bond [59,60]. Specifically, charge difference analysis was employed to quantify the redistribution of charge on the semiconductor surface resulting from the adsorption of cyanide species [60]. Additionally, the electron localization function (ELF) was utilized to gain deeper insights into the nature of the cyanide–semiconductor interaction [64]. In ELF analysis, the distribution of maximum electron density (referred to as the Region of Maximum Density or RMD) around the atomic nucleus provides information on the nature of the interaction. When RMD is symmetrically distributed around the nucleus, it indicates a more ionic or van der Waals interaction. Conversely, as the covalent character of a bond increases, the migration of RMD between atomic centers becomes more pronounced until an ideal covalent interaction is achieved, characterized by complete symmetry [65].

### 4.5. Photodegradation Studies

The heterogeneous photocatalysis experiments were conducted following the methodology outlined in our prior investigation [74]. These experiments were carried out under UVB irradiation conditions. The batch approach was employed, and the solution pH was maintained at 10 ± 0.1 by the addition of 0.1 M hydrochloric acid or sodium hydroxide solutions. Typically, semiconductor nanoparticles were subjected to magnetic stirring in a 500 mL aqueous KCN solution (100 mg L^−1^) containing 0.1–1.0% hydrogen peroxide (H_2_O_2_). The degradation rate of cyanide species in the heterogeneous photocatalytic systems exposed to UVB light was monitored using the Langmuir–Hinshelwood equation [98,99], which is expressed by Equation (19) as presented in Table 1.

### 4.6. Reuse of Nanoparticles

To assess the reusability of the nanoparticles in the degradation of cyanide, a recycling experiment was devised, following the methodology delineated in our prior study [74]. This recycling experiment was carried out over five consecutive cycles. After the completion of each treatment cycle, the suspensions were allowed to settle for a minimum of 1 h. Following this settling period, the supernatant liquid was decanted, and the nanoparticles were meticulously washed three times with HPLC-grade water to prevent any loss of solid material. In each treatment cycle, 500 mL of a fresh KCN solution (100 mg L^−1^) was employed, with the nanoparticle concentration set at 0.2 g L^−1^ and the H_2_O_2_ concentration at 0.1% (*v*/*v*).

### 4.7. Toxicity Studies

Bioassay tests are essential for evaluating the suitability of synthesized materials in water treatment applications. Artemia salina, commonly referred to as brine shrimp, serves as a pertinent model organism in toxicological and ecotoxicity studies of nanoparticles and aqueous systems [100], with its usage endorsed by the United States Environmental Protection Agency (US-EPA) for acute toxicity assessments [101].

In conducting the bioassay with Artemia salina, an adapted method described by previous researchers was employed [102]. In a glass container, a 1 L solution of artificial seawater was prepared according to the Dietrich and Kalle formula, which comprises distilled water, NaCl, MgCl_2_∙6H_2_O, Na_2_SO_4_, CaCl_2_∙2H_2_O, KCl, and the pH was adjusted to 9.0 with Na_2_CO_3_ [103]. For hatching Artemia salina cysts, 500 mL of this artificial seawater was utilized, shielded with aluminum foil except for a small opening on the upper surface exposed to illumination from a 20 W lamp situated approximately 32 cm away. Oxygenation was ensured for 15 min before introducing the cysts, and continuous aeration was maintained throughout the incubation period at a temperature of 28 °C, with a salinity of 32 μg mL^−1^ and a pH of 9.0. Consistent oxygenation/aeration was upheld to prevent cyst agglutination.

To assess acute toxicity, cyanide solutions treated with the most efficient removal system at various time intervals were diluted in artificial seawater. The negative control consisted of artificial seawater without the test substance (cyanide), while the positive control comprised artificial seawater with an untreated cyanide solution. The bioassay was organized in test tubes, each containing 10 mL of the test or control solution, to which 10 nauplii were introduced and incubated for 48 h at 25 °C. After 24 and 48 h, the number of live nauplii was quantified, and any aberrant behavior, such as swimming difficulties, was noted. All tests were conducted in triplicate. 

## 5. Conclusions

In this study, the use of a ZnTiO_3_/2/H_2_O_2_/UVB system for cyanide removal is described for the first time. The experimental results were complemented by theoretical studies. Both practical results and in silico results demonstrate that it is possible to remove cyanide ion and hydrogen cyanide from aqueous solutions using ZnTiO_3_/TiO_2_ nanoparticles in a system that includes UVB radiation and hydrogen peroxide. The amount of hydrogen peroxide used was minimized, in order to reduce its impact when using this system on an industrial scale. The results presented in this research can be used as a basis for new studies with other contaminants. For future studies, it is proposed to test the ZnTiO_3/_TiO_2_/H_2_O_2_/UVB system on a pilot scale and/or with other contaminants.

Finally, from a molecular perspective, the hybrid semiconductor material ZnTiO_3_/TiO_2_ is justified as a highly effective choice for cyanide remediation through adsorption and photocatalysis. Its unique molecular properties, including its adsorption and photocatalysis capacity, as well as its charge transfer capacity, enable the efficient and complete removal of cyanide from contaminated wastewater. Adsorption facilitates the capture and retention of cyanide on the surface of the material, while photocatalysis and charge transfer reactions promote its degradation into non-toxic compounds. These molecular mechanisms strongly support the choice of ZnTiO_3_/TiO_2_ as a material for the design of advanced treatment systems aimed at cyanide remediation. This enriched molecular understanding lays the foundation for future research and applications in the efficient removal of cyanide contaminants from the environment.

## Figures and Tables

**Table 1:** Summary of the mathematical equations used in this study.

Denomination	Equation		Parameters
Scherrer equation	$A=\frac{K\lambda }{\beta cos\theta }$	(1)	λ = Wavelength of the X-ray beam (0.15406 nm) K = Shape factor (0.89) θ = Bragg angle β = Full width at half peak height maximum (FWHM) of the X-ray diffraction peak
Adsorbate adsorbed	$q_{e}=\left(C_{0}-C_{e}\right)\times \frac{v}{w}$	(2)	C_0_ = Initial concentration (mg L^−1^) Ce = Equilibrium concentration (mg L^−1^) w = Mass of the adsorbent (g) v = Volume of the solution (L)
Langmuir	$\frac{C_{e}}{q_{e}}=\frac{1}{K_{L}q_{max}}+\frac{C_{e}}{q_{max}}$	(3)	q_max_ = Maximum monolayer adsorption (mg g^−1^) K_L_ = Equilibrium Langmuir constant related to the adsorption energy (L mg^−1^) C_e_ = Concentration of adsorbate in solution at equilibrium (mg L^−1^)
Freundlich	qe=KFCe1n	(4)	K_F_ = Freundlich constant (L mg^−1^) 1/n = Adsorption intensity constant. Note: For favorable adsorption, the value of n should be between 1 and 10
Temkin	$q_{e}=Bln(AC_{e})$	(5)	q_e_ = Adsorbate adsorbed per unit weight (mg g^−1^) at equilibrium A = Temkin isotherm constant (L g^−1^) C_e_ = Concentration of adsorbate in solution at equilibrium (mg L^−1^) B = Constant related to the heat adsorption
Constant of heat adsorption	$B=\frac{RT}{b}$	(6)	b = Temkin constant (J mol^−1^) T = Absolute temperature (K) R = Gas constant (8.314 J mol^−1^ K^−1^)
Separation factor	$R_{L}=\frac{1}{\left(1+K_{L}C_{e}\right)}$	(7)	K_L_ = Equilibrium Langmuir constant related to the adsorption energy (L mg^−1^) C_e_ = Concentration of adsorbate in solution at equilibrium (mg L^−1^) Note: 0 < R_L_ < 1, suitable adsorption, R_L_ > 1 suitable adsorption, R_L_ = 0 irreversible adsorption, R_L_ = 1 linear adsorption.
Gibbs free energy	$∆G^{0}=-RT\;ln\;k_{C}$	(8)	∆G^0^ = Gibbs free energy (kJ mol^−1^), ∆H^0^ = Enthalpy (kJ mol^−1^) ∆S^0^ = Entropy (kJ mol^−1^ K^−1^)
Van’t Hoff equation	$\ln k_{C}=\frac{-∆H^{0}}{R}\times \frac{1}{T}+\frac{∆S^{0}}{R}$	(9)	k_C_ = Dimensionless parameter T = Absolute temperature (K) R = Universal gas constant (8.314 J mol^−1^ K^−1^)
	$k_{C}=k_{L}\times M_{w}\times 1000\times 55.5$	(10)	k_L_ = Langmuir constant (L mg^−1^) M_w_ = Adsorbate weight (g mol^−1^)
Pseudo first order	$\ln\left(q_{e}-q_{t}\right)=\ln\left(q_{e}\right)-k_{1}t$	(11)	k_1_ = Rate constant (min^−1^) q_e_ = Adsorbate adsorbed per unit weight (mg g^−1^) at equilibrium q_t_ = Adsorbate adsorbed per unit weight (mg g^−1^) at any time (t)
Pseudo second order	$\frac{t}{q_{t}}=\frac{1}{k_{2}q_{e}^{2}}+\frac{1}{q_{e}}t$	(12)	k_2_ = Rate constant (g mg^−1^ min^−1^) q_e_ = Adsorbate adsorbed per unit weight (mg g^−1^) at equilibrium q_t_ = Adsorbate adsorbed per unit weight (mg g^−1^) at any time (t)
Elovich	$q_{t}=\frac{1}{\beta }\ln\left(\alpha \beta \right)+\frac{1}{\beta }ln(t)$	(13)	q_t_ = Adsorbate adsorbed per unit weight (mg g^−1^) at any time (t) α = Constant related to chemisorption rate β = Constant which depicts the extent of surface coverage
Intraparticle-diffusion	qt=k3t12+A	(14)	k_3_ = Intraparticle diffusion rate constant (mg g^−1^ min^−1/2^) A = constant indicating the width of the boundary layer (mg g^−1^). The larger the value of A, the greater the boundary layer effect.
Particle-diffusion	$-ln\left(1-{\left(\frac{q_{t}}{q_{e}}\right)}^{2}\right)=\frac{2\pi^{2}D_{p}}{r^{2}}t$	(15)	q_e_ = Adsorbate adsorbed per unit weight (mg g^−1^) at equilibrium q_t_ = Adsorbate adsorbed per unit weight (mg g^−1^) at any time (t) *C*_z_ = Ion concentration o the adsorbent (mg kg^−1^). D_p_ = Diffusion coefficient in the adsorbent phase (m^2^ min^−1^) r = Average radius of the adsorbent particles (1 × 10^−7^ m) t = Contact time (min)
External-film-diffusion	$-ln\left(1-\left(\frac{q_{t}}{q_{e}}\right)\right)=\frac{D_{f}C_{s}}{hrC_{z}}t$	(16)	q_e_ = Adsorbate adsorbed per unit weight (mg g^−1^) at equilibrium q_t_ = Adsorbate adsorbed per unit weight (mg g^−1^) at any time (t) D_f_ = Diffusion in the film phase surrounding the adsorbent particles (m^2^ min^−1^) *C*_s_ = Ion concentration in the solution (mg L^−1^) h = Film thickness around the adsorbent particles (10^−6^ m in poorly stirred solutions) r = Average radius of the adsorbent particles (1 × 10^−7^ m) t = Contact time (min)
Surface energy	$\gamma_{s}=\frac{\left(E_{slab}-n\times E_{bulk}\right)}{2A}$	(17)	E_slab_ = Total energy of the slab material (eV) E_bulk_ = Total energy of the bulk material (eV) n = Number of atoms involved in the slab A = Surface area (Å^2^)
Adsorption energy	$E_{ads}=E_{sorb/surf}-E_{surf}-E_{sorb}$	(18)	E_sorb/surf_ = Energy of the supersystem produced by the adsorbed molecule on the surface (eV) E_surf_ = Energy of the surface (eV) E_sorb_ = Energy of the isolated molecule in vacuum (eV)
Langmuir-Hinshelwood equation	$ln\frac{C_{o}}{C_{t}}=kKt=k_{app}t$	(19)	k = Actual rate constant (min^−1^) K = Adsorption constant of the substrate on the nanoparticles C_0_ = Initial concentration of the substrate (mg L^−1^) C_t_ = concentration at a specific time (mg L^−1^) k_app_ = apparent rate constant (min^−1^)

**Table 2:** Elemental composition of TiO_2_ and ZnTiO_3_/TiO_2_ nanoparticles.

Element	TiO_2_		ZnTiO_3_/TiO_2_	
	Weight %	Atomic %	Weight %	Atomic %
C	6.15	14.36	5.42	11.90
O	26.31	46.10	33.60	55.41
Ti	67.54	39.54	54.85	30.22
Zn	–	–	6.13	2.47

**Table 3:** Effect of the composition of the reaction system on the cyanide removal capacity.

Reaction System Composition		HSD Tukey *	Duncan *
ZnTiO_3_/TiO_2_ (mg L^−1^)	H_2_O_2_ (%)	q_e_ (mg g^−1^)	q_e_ (mg g^−1^)
200	1.00	185.56 ± 2.95 ^a^	185.56 ± 2.95 ^a^
200	0.50	176.00 ± 2.18 ^a,b^	176.00 ± 2.18 ^b^
200	0.25	145.64 ± 2.46 ^b,c^	154.64 ± 2.46 ^c^
200	0.10	162.02 ± 2.62 ^d,e,f^	162.02 ± 2.62 ^d,e^
150	1.00	164.08 ± 2.12 ^b,c^	164.08 ± 2.12 ^c^
150	0.50	158.24 ± 2.23 ^c,d^	158.24 ± 2.23 ^c^
150	0.25	149.47 ± 2.59 ^c,d,e^	149.47 ± 2.59 ^d^
150	0.10	137.54 ± 2.31 ^e,f,g^	137.54 ± 2.31 ^e,f^
75	1.00	134.62 ± 2.16 ^e,f,g^	134.62 ± 2.16 ^f,g^
75	0.50	131.92 ± 2.77 ^f,g^	131.92 ± 2.77 ^f,g^
75	0.25	126.52 ± 2.23 ^g^	126.52 ± 2.23 ^g^
75	0.10	107.63 ± 2.39 ^h^	107.63 ± 2.39 ^h^
20	1.00	98.41 ± 2.31 ^h^	98.41 ± 2.31 ^i^
20	0.50	82.22 ± 2.16 ^i^	82.22 ± 2.16 ^j^
20	0.25	75.02 ± 2.67 ^i^	75.02 ± 2.67 ^j^
20	0.10	44.43 ± 2.47 ^j^	44.43 ± 2.47 ^k^
*p*-value		<0.001	<0.001

The means for the groups in the homogeneous subsets are displayed. * Use the sample size of the harmonic mean = 3.0.

**Table 4:** Isotherm parameters for cyanide sorption on nanoparticles at different temperatures.

Isotherm Parameters		293.15 K	298.15 K	303.15 K
Langmuir	q_max_ (mg g^−1^)	74.49 (±2.42)	81.94 (±1.52)	89.39 (±1.52)
	K_L_ (L mg^−1^)	0.27 (±0.01)	0.32 (±0.04)	0.36 (±0.03)
	R_L_	0.16	0.14	0.36
	χ^2^	2.65	3.32	2.97
	R^2^	0.99	1.00	0.98
Freundlich	K_F_ (L mg^−1^)	12.15 (±2.36)	13.36 (±1.94)	14.58 (±1.74)
	n	2.46 (±0.32)	2.71 (±0.39)	2.96 (±0.39)
	1/n	0.41	0.37	0.34
	χ^2^	2.89	3.21	2.45
	R^2^	0.94	0.97	0.95
Temkin	B	13.90 (±1.03)	15.30 (±1.94)	16.82 (±1.89)
	A	1.32 (±0.30)	1.46 (±0.39)	1.60 (±0.35)
	χ^2^	2.39	3.17	2.86
	R^2^	0.96	0.98	0.96

**Table 5:** Thermodynamic parameters of the cyanide adsorption onto ZnTiO_3_/TiO_2_ nanoparticles.

Temperature (K)	ln k_C_	∆G° (kJ mol^−1^)	∆H° (kJ mol^−1^)	∆S° (kJ mol^−1^ K^−1^)
293.15	12.91	−31.47	21.27	0.18
298.15	13.08	−32.43		
303.15	13.20	−33.27		

**Table 6:** Kinetic parameters for cyanide adsorption onto ZnTiO_3_/TiO_2_ nanoparticles.

Kinetic Parameters		ZTO/TO
Pseudo-first-order	q_max_ (mg g^−1^)	201.79 (±1.61)
	k_1_ (L mg^−1^)	0.06 (±1.60 × 10^−3^)
	χ^2^	6.18
	R^2^	0.98
Pseudo-second-order	q_max_ (mg g^−1^)	236.64 (±1.81)
	k_2_ (L mg^−1^)	2.86 × 10^−4^ (±4.33 × 10^−5^)
	χ^2^	7.41
	R^2^	0.99
Elovich	α	34.35 (±7.06)
	β	0.02 (±1.74 × 10^−3^)
	χ^2^	6.82
	R^2^	0.95
Intraparticle diffusion	k_3_ (mg g^−1^ min^−1/2^)	18.01 (±0.58)
	A	39.17 (±1.35)
	R^2^	0.84
External-film diffusion	D*f* (m^2^ min^−1^)	1.40 × 10^−11^
	R^2^	0.98
Internal-pore diffusion	D*p* (m^2^ min^−1^)	4.60 × 10^−17^
	R^2^	0.92

**Table 7:** Bader charge (e) analysis for atoms interacting in adsorption.

Atom	CN	HCN	TiO_2_	CN-TiO_2_	HCN-TiO_2_	ZnTiO_3_	CN-ZnTiO_3_	HCN-ZnTiO_3_
H1	-	+0.21	-	-	+0.24	-	-	+0.29
C1	+3.96	+2.35	-	+4.00	+2.37	-	+4.00	+2.30
N1	−3.96	−2.56	-	−2.80	−2.61	-	−2.77	−2.60
Ti57	-	-	+2.54	+2.35	+0.02	-	-	-
Ti60	-	-	+2.56	+2.48	+0.00	-	-	-
O59	-	-	−1.16	−2.09	−0.04	-	-	-
Ti23	-	-	-	-	-	+2.51	+2.36	+0.01
O8	-	-	-	-	-	−1.11	−2.04	−1.15

**Table 8:** Apparent rate constant (k_app_) for photodegradation of cyanide species.

Description	k_app_ (min^−1^)
ZnTiO_3_/TiO_2_ (200 mg L^−1^)/H_2_O_2_ (0.10%)	0.0738
ZnTiO_3_/TiO_2_ (150 mg L^−1^)/H_2_O_2_ (0.25%)	0.0395
ZnTiO_3_/TiO_2_ (75 mg L^−1^)/H_2_O_2_ (0.50%)	0.0306
ZnTiO_3_/TiO_2_ (20 mg L^−1^)/H_2_O_2_ (1.0%)	0.0246

**Table 9:** Results of the photocatalytic degradation experiment.

No.	H_2_O_2_ (%)	ZnTiO_3_/TiO_2_ (mg L^−1^)	UVB Light	Removal (%)
1	1	200	Absent	75.46
2	1	150	Absent	64.71
3	1	75	Absent	54.40
4	1	20	Absent	42.71
5	1	20	Present	78.48
6	0.5	200	Absent	71.47
7	0.5	150	Absent	63.93
8	0.5	75	Absent	53.58
9	0.5	20	Absent	36.08
10	0.5	75	Present	85.26
11	0.25	200	Absent	69.83
12	0.25	150	Absent	60.99
13	0.25	75	Absent	51.72
14	0.25	20	Absent	29.53
15	0.25	150	Present	92.22
16	0.1	200	Absent	67.95
17	0.1	150	Absent	55.56
18	0.1	75	Absent	44.33
19	0.1	20	Absent	25.20
20	0.1	200	Present	99.74
21	0.1	0	Absent	12.63
22	0	20	Absent	18.17
23	0	0	Present	0.00

## Data Availability

Data are contained within the article.

## Supplementary Materials

The following supporting information can be downloaded at: https://www.mdpi.com/article/10.3390/ijms242216446/s1.

## References

[1] Anandan S., Kumar Ponnusamy V., Ashokkumar M. (2020). A review on hybrid techniques for the degradation of organic pollutants in aqueous environment. Ultrason. Sonochem..

[2] Alvillo-Rivera A., Garrido-Hoyos S., Buitrón G., Thangarasu-Sarasvathi P., Rosano-Ortega G. (2021). Biological treatment for the degradation of cyanide: A review. J. Mater. Res. Technol..

[3] Pan Y., Zhang Y., Huang Y., Jia Y., Chen L., Cui H. (2021). Synergistic effect of adsorptive photocatalytic oxidation and degradation mechanism of cyanides and Cu/Zn complexes over TiO_2_/ZSM-5 in real wastewater. J. Hazard. Mater..

[4] Maciel A.C., da Silva Pena R., do Nascimento L.D., de Oliveira T.A., Chagas-Junior G.C.A., Lopes A.S. (2023). Health exposure risks and bioremediation of cyanide in cassava processing effluents: An overview. J. Water Process Eng..

[5] Betancourt-Buitrago L.A., Hernandez-Ramirez A., Colina-Marquez J.A., Bustillo-Lecompte C.F., Rehmann L., Machuca-Martinez F. (2019). Recent Developments in the Photocatalytic Treatment of Cyanide Wastewater: An Approach to Remediation and Recovery of Metals. Processes.

[6] Biswas P., Bhunia P., Saha P., Sarkar S., Chandel H., De S. (2020). In situ photodecyanation of steel industry wastewater in a pilot scale. Environ. Sci. Pollut. Res..

[7] Zhang Y., Zhang Y., Huang Y., Chen X., Cui H., Wang M. (2020). Enhanced photocatalytic reaction and mechanism for treating cyanide-containing wastewater by silicon-based nano-titania. Hydrometallurgy.

[8] Han W., Yang H., Tong L. (2022). Cyanide removal for ultrafine gold cyanide residues by chemical oxidation methods. Trans. Nonferrous Met. Soc. China.

[9] Wei Y., Chen L., Jiao G., Wen Y., Liao Q., Zhou H., Tang S. (2023). Enhanced removal of metal-cyanide complexes from wastewater by Fe-impregnated biochar: Adsorption performance and removal mechanism. Chemosphere.

[10] Yagmur Goren A., Recepoglu Y.K., Yoon Y., Khataee A. (2023). Insights into sustainability of engineered carbonaceous material-based technologies for advanced cyanide removal from wastewater. Alex. Eng. J..

[11] Kundu A., Cherwoo L., Kumar B. (2023). Leveraging microorganisms for phenol and cyanide degradation in coke oven industry effluent treatment: Current advances and future potential. Bioresour. Technol. Reports.

[12] Kim T.K., Kim T., Jo A., Park S., Choi K., Zoh K.D. (2018). Degradation mechanism of cyanide in water using a UV-LED/H_2_O_2_/Cu^2+^ system. Chemosphere.

[13] Zmirli Z., Driouich A., El Harfaoui S., Mohssine A., Chaair H., Sallek B. (2023). Cyanide effluent treatment by electrocoagulation using airlift reactor: Modeling and optimization by response surface methodology. Sci. Afr..

[14] Thaweewong P., Chotineeranat S., Anuntagool J. (2023). Removal of free cyanide in dry-milled cassava flour using atmospheric nonthermal plasma treatment. LWT.

[15] Pan Y., Zhang Y., Huang Y., Jia Y., Chen L., Cui H. (2022). Functional Ag-doped coralloid titanosilicate zeolite (CTS-Ag) for efficiently catalytic and photodegradative removal of free cyanides and copper/zinc-cyanide complexes in real wastewater. J. Alloys Compd..

[16] Dong K., Xie F., Wang W., Chang Y., Lu D., Gu X., Chen C. (2021). The detoxification and utilization of cyanide tailings: A critical review. J. Clean. Prod..

[17] Eletta O.A.A., Ajayi O.A., Ogunleye O.O., Akpan I.C. (2016). Adsorption of cyanide from aqueous solution using calcinated eggshells: Equilibrium and optimisation studies. J. Environ. Chem. Eng..

[18] Núñez-Salas R.E., Hernández-Ramírez A., Hinojosa-Reyes L., Guzmán-Mar J.L., Villanueva-Rodríguez M., de Lourdes Maya-Treviño M. (2019). Cyanide degradation in aqueous solution by heterogeneous photocatalysis using boron-doped zinc oxide. Catal. Today.

[19] Chen X., Ren Y., Qu G., Wang Z., Yang Y., Ning P. (2023). A review of environmental functional materials for cyanide removal by adsorption and catalysis. Inorg. Chem. Commun..

[20] Maulana I., Takahashi F. (2018). Cyanide removal study by raw and iron-modified synthetic zeolites in batch adsorption experiments. J. Water Process Eng..

[21] Boczkaj G., Fernandes A. (2017). Wastewater treatment by means of advanced oxidation processes at basic pH conditions: A review. Chem. Eng. J..

[22] Singh S., Kumar V., Datta S., Dhanjal D.S., Sharma K., Samuel J., Singh J. (2020). Current advancement and future prospect of biosorbents for bioremediation. Sci. Total Environ..

[23] Lincho J., Zaleska-Medynska A., Martins R.C., Gomes J. (2022). Nanostructured photocatalysts for the abatement of contaminants by photocatalysis and photocatalytic ozonation: An overview. Sci. Total Environ..

[24] Liu Q., Wu Z., Sun Z., Wang Q., Shi J. (2023). Enhanced natural degradation of cyanide tailings: Integrated application of solar drying system and UV irradiation. J. Hazard. Mater..

[25] Ma Y., Sun H., Wang Q., Sun L., Liu Z., Xie Y., Zhang Q., Su C., Fan D. (2023). Driving hydrogen peroxide artificial photosynthesis and utilization for emerging contaminants removal by cyanided polymeric carbon nitride. Appl. Catal. B Environ..

[26] Chuaicham C., Karthikeyan S., Song J.T., Ishihara T., Ohtani B., Sasaki K. (2020). Importance of ZnTiO_3_ Phase in ZnTi-Mixed Metal Oxide Photocatalysts Derived from Layered Double Hydroxide. ACS Appl. Mater. Interfaces.

[27] Zhang L., Djellabi R., Su P., Wang Y., Zhao J. (2023). Through converting the surface complex on TiO_2_ nanorods to generate superoxide and singlet oxygen to remove CN−. J. Environ. Sci..

[28] Coronel S., Endara D., Lozada A.B., Manangón-Perugachi L.E., de la Torre E. (2021). Photocatalytic study of cyanide oxidation using titanium dioxide (TiO_2_)-activated carbon composites in a continuous flow photo-reactor. Catalysts.

[29] Sánchez-Tovar R., Blasco-Tamarit E., Fernández-Domene R.M., Villanueva-Pascual M., García-Antón J. (2020). Electrochemical formation of novel TiO_2_-ZnO hybrid nanostructures for photoelectrochemical water splitting applications. Surf. Coat. Technol..

[30] Al-Mamun M.R., Kader S., Islam M.S., Khan M.Z.H. (2019). Photocatalytic activity improvement and application of UV-TiO_2_ photocatalysis in textile wastewater treatment: A review. J. Environ. Chem. Eng..

[31] Kanakaraju D., Chandrasekaran A. (2023). Recent advances in TiO_2_/ZnS-based binary and ternary photocatalysts for the degradation of organic pollutants. Sci. Total Environ..

[32] Dubsok A., Khamdahsag P., Kittipongvises S. (2022). Life cycle environmental impact assessment of cyanate removal in mine tailings wastewater by nano-TiO_2_/FeCl_3_ photocatalysis. J. Clean. Prod..

[33] Suhan M.B.K., Al-Mamun M.R., Farzana N., Aishee S.M., Islam M.S., Marwani H.M., Hasan M.M., Asiri A.M., Rahman M.M., Islam A. (2023). Sustainable pollutant removal and wastewater remediation using TiO_2_-based nanocomposites: A critical review. Nano-Struct. Nano-Objects.

[34] Lin L., Yang Y., Men L., Wang X., He D., Chai Y., Zhao B., Ghoshroy S., Tang Q. (2013). A highly efficient TiO_2_@ZnO n-p-n heterojunction nanorod photocatalyst. Nanoscale.

[35] Fu R., Wang Q., Gao S., Wang Z., Huang B., Dai Y., Lu J. (2015). Effect of different processes and Ti/Zn molar ratios on the structure, morphology, and enhanced photoelectrochemical and photocatalytic performance of Ti_3_^+^ self-doped titanium-zinc hybrid oxides. J. Power Sources.

[36] Pengkalsinan K., Tio Z., Melalui F. (2017). Effect of calcination temperature on ZnO/TiO_2_ composite in photocatalytic treatment of phenol under visible light. Malays. J. Anal. Sci..

[37] Delsouz Khaki M.R., Shafeeyan M.S., Raman A.A.A., Daud W.M.A.W. (2018). Enhanced UV–Visible photocatalytic activity of Cu-doped ZnO/TiO_2_ nanoparticles. J. Mater. Sci. Mater. Electron..

[38] Khang K.C.L., Hatta M.H.M., Lee S.L., Yuliati L. (2018). Photocatalytic removal of phenol over mesoporous ZnO/TiO_2_ composites. J. Teknol..

[39] Chorf H., Saadoun M., Bousselmi L., Bessais B., Chorfi H., Saadoun M., Bousselmi L., Bessais B. (2012). TiO_2_-ITO and TiO_2_-ZnO nanocomposites: Application on water treatment. EPJ Web of Conferences.

[40] Stefańska K.S., Kubiaka A., Piasecki A., Goscianska J., Nowaczyk G., Jurga S., Jesionowski T. (2018). TiO_2_-ZnO Binary Oxide Systems: Comprehensive Characterization and Tests of Photocatalytic Activity. Materials.

[41] Jaramillo-Fierro X., González S., Montesdeoca-Mendoza F., Medina F. (2021). Structuring of ZnTiO_3_/TiO_2_ adsorbents for the removal of methylene blue, using zeolite precursor clays as natural additives. Nanomaterials.

[42] Jaramillo-Fierro X., Alvarado H., Montesdeoca F., Valarezo E. (2023). Faujasite-Type Zeolite Obtained from Ecuadorian Clay as a Support of ZnTiO_3_/TiO_2_ NPs for Cyanide Removal in Aqueous Solutions. Int. J. Mol. Sci..

[43] Cosmos A., Erdenekhuyag B.O., Yao G., Li H., Zhao J., Laijun W., Lyu X. (2020). Principles and methods of bio detoxification of cyanide contaminants. J. Mater. Cycles Waste Manag..

[44] Ke S., Cheng X., Wang Q., Wang Y., Pan Z. (2014). Preparation of a photocatalytic TiO_2_/ZnTiO_3_ coating on glazed ceramic tiles. Ceram. Int..

[45] Ramgir N., Bhusari R., Rawat N.S., Patil S.J., Debnath A.K., Gadkari S.C., Muthe K.P. (2020). TiO_2_/ZnO heterostructure nanowire based NO_2_ sensor. Mater. Sci. Semicond. Process..

[46] Gnanaseelan N., Latha M., Mantilla A., Sathish-Kumar K., Caballero-Briones F. (2020). The role of redox states and junctions in photocatalytic hydrogen generation of MoS_2_-TiO_2_-rGO and CeO_2_-Ce_2_Ti_3_O_8_.7-TiO_2_-rGO composites. Mater. Sci. Semicond. Process..

[47] Amaro-Medina B.M., Martinez-Luevanos A., de Jesus Soria-Aguilar M., Sanchez-Castillo M.A., Estrada-Flores S., Carrillo-Pedroza F.R. (2022). Efficiency of Adsorption and Photodegradation of Composite TiO_2_/Fe_2_O_3_ and Industrial Wastes in Cyanide Removal. Water.

[48] Stavropoulos G.G., Skodras G.S., Papadimitriou K.G. (2015). Effect of solution chemistry on cyanide adsorption in activated carbon. Appl. Therm. Eng..

[49] Muthirulan P., Nirmala Devi C., Meenakshi Sundaram M. (2017). Synchronous role of coupled adsorption and photocatalytic degradation on CAC–TiO_2_ composite generating excellent mineralization of alizarin cyanine green dye in aqueous solution. Arab. J. Chem..

[50] Holzwarth U., Gibson N. (2011). The Scherrer equation versus the “Debye-Scherrer equation”. Nat. Nanotechnol..

[51] Kosmulski M. (2018). The pH dependent surface charging and points of zero charge. VII. Update. Adv. Colloid Interface Sci..

[52] Ramakrishnan V.M., Natarajan M., Santhanam A., Asokan V., Velauthapillai D. (2018). Size controlled synthesis of TiO_2_ nanoparticles by modified solvothermal method towards effective photo catalytic and photovoltaic applications. Mater. Res. Bull..

[53] Rueden C.T., Schindelin J., Hiner M.C., DeZonia B.E., Walter A.E., Arena E.T., Eliceiri K.W. (2017). ImageJ2: ImageJ for the next generation of scientific image data. BMC Bioinform..

[54] Schneider C.A., Rasband W.S., Eliceiri K.W. (2012). NIH Image to ImageJ: 25 years of image analysis. Nat. Methods.

[55] Vorontsov A.V., Tsybulya S.V. (2018). Influence of Nanoparticles Size on XRD Patterns for Small Monodisperse Nanoparticles of CuO and TiO_2_ Anatase. Ind. Eng. Chem. Res..

[56] Aliprandini P., Botelho A.B., Veiga M.M., Marshall B.G., Scarazzato T., Espinosa D.C.R. (2023). Evaluation of biosorbents as an alternative for mercury cyanide removal from aqueous solution. Miner. Eng..

[57] Jaramillo-Fierro X., Capa L.F., Medina F., González S. (2021). Dft study of methylene blue adsorption on ZnTiO_3_ and TiO_2_ surfaces (101). Molecules.

[58] Lin C.S., Zhou A.Y., Cheng W.D., Ye N., Chai G.L. (2019). Atom-Resolved Analysis of Birefringence of Nonlinear Optical Crystals by Bader Charge Integration. J. Phys. Chem. C.

[59] Kumar P.S.V., Raghavendra V., Subramanian V. (2016). Bader’s Theory of Atoms in Molecules (AIM) and its Applications to Chemical Bonding. J. Chem. Sci..

[60] Zhang H., Huang W., Wang W.C., Shi X.Q. (2018). Ionicity of bonding in elemental solids. J. Phys. Commun..

[61] Jaramillo-Fierro X., Gaona S., Valarezo E. (2022). La^3+^’s Effect on the Surface (101) of Anatase for Methylene Blue Dye Removal, a DFT Study. Molecules.

[62] Jaramillo-Fierro X., Cuenca G., Ramón J. (2022). The Effect of La_3+_ on the Methylene Blue Dye Removal Capacity of the La/ZnTiO_3_ Photocatalyst, a DFT Study. Nanomaterials.

[63] Koch D., Golub P., Manzhos S. (2018). Stability of charges in titanium compounds and charge transfer to oxygen in titanium dioxide. J. Phys. Conf. Ser..

[64] Savin A., Nesper R., Wengert S., Fässler T.F. (1997). ELF: The Electron Localization Function. Angew. Chemie Int. Ed. Engl..

[65] Wen C., Zhu Y.J., Kanbara T., Zhu H.Z., Xiao C.F. (2009). Effects of I and F codoped TiO_2_ on the photocatalytic degradation of methylene blue. Desalination.

[66] Bishayee B., Rai A., Kumar A., Kamila B., Ruj B., Dutta S. (2023). End-of-pipe treatment of secondary treated coke-oven wastewater for removal of fluoride, cyanide, phenol, ammoniacal-N and nitrate using waste material: Experiment, modelling and optimization. Chem. Eng. Res. Des..

[67] Dash R.R., Gaur A., Balomajumder C., Roshan R., Gaur A., Balomajumder C. (2009). Cyanide in industrial wastewaters and its removal: A review on biotreatment. J. Hazard. Mater..

[68] Gupta N., Balomajumder C., Agarwal V.K. (2012). Adsorption of cyanide ion on pressmud surface: A modeling approach. Chem. Eng. J..

[69] Saxena S., Prasad M., Amritphale S.S., Chandra N. (2001). Adsorption of cyanide from aqueous solutions at pyrophyllite surface. Sep. Purif. Technol..

[70] Benmessaoud A., Nibou D., Mekatel E.H., Amokrane S. (2020). A Comparative Study of the Linear and Non-Linear Methods for Determination of the Optimum Equilibrium Isotherm for Adsorption of Pb_2_^+^ Ions onto Algerian Treated Clay. Iran. J. Chem. Chem. Eng..

[71] Guo H., Chen J., Weng W., Zheng Z., Wang D. (2014). Adsorption behavior of Congo red from aqueous solution on La_2_O_3_-doped TiO_2_ nanotubes. J. Ind. Eng. Chem..

[72] Gil A., Assis F.C.C., Albeniz S., Korili S.A. (2011). Removal of dyes from wastewaters by adsorption on pillared clays. Chem. Eng. J..

[73] Zhang J., Xu B., Wang Y.S., Qin Z., Ke S.H. (2019). First-principles investigation of the ferroelectric, piezoelectric and nonlinear optical properties of LiNbO_3_-type ZnTiO_3_. Sci. Rep..

[74] Jaramillo-Fierro X., González S., Medina F. (2021). La-doped ZnTiO_3_/TiO_2_ nanocomposite supported on ecuadorian diatomaceous earth as a highly efficient photocatalyst driven by solar light. Molecules.

[75] Bakatula E.N., Richard D., Neculita C.M., Zagury G.J. (2018). Determination of point of zero charge of natural organic materials. Environ. Sci. Pollut. Res..

[76] Wang Z., Yuan T., Yao J., Li J., Jin Y., Cheng J., Shen Z. (2021). Development of an unmanned device with picric acid strip for on-site rapid detections of sodium cyanide in marine water. IOP Conf. Ser. Earth Environ. Sci..

[77] Pramitha A.R., Harijono H., Wulan S.N. (2021). Comparison of cyanide content in arbila beans (*Phaseolus lunatus* L.) of East Nusa Tenggara using picrate and acid hydrolysis methods. IOP Conf. Ser. Earth Environ. Sci..

[78] Castada H.Z., Liu J., Barringer S.A., Huang X. (2020). Cyanogenesis in Macadamia and Direct Analysis of Hydrogen Cyanide in Macadamia Flowers, Leaves, Husks, and Nuts Using Selected Ion Flow Tube–Mass Spectrometry. Foods.

[79] Eke-emezie N., Etuk B.R., Akpan O.P., Chinweoke O.C. (2022). Cyanide removal from cassava wastewater onto H_3_PO_4_ activated periwinkle shell carbon. Appl. Water Sci..

[80] Pirmoradi M., Hashemian S., Shayesteh M.R. (2017). Kinetics and thermodynamics of cyanide removal by ZnO@NiO nanocrystals. Trans. Nonferrous Met. Soc. China (Engl. Ed.).

[81] Noroozi R., Al-Musawi T.J., Kazemian H., Kalhori E.M., Zarrabi M. (2018). Removal of cyanide using surface-modified Linde Type-A zeolite nanoparticles as an efficient and eco-friendly material. J. Water Process Eng..

[82] Inyinbor A.A., Adekola F.A., Olatunji G.A. (2016). Kinetics, isotherms and thermodynamic modeling of liquid phase adsorption of Rhodamine B dye onto Raphia hookerie fruit epicarp. Water Resour. Ind..

[83] Tran H.N., You S.J., Hosseini-Bandegharaei A., Chao H.P. (2017). Mistakes and inconsistencies regarding adsorption of contaminants from aqueous solutions: A critical review. Water Res..

[84] Zhou X., Zhou X. (2014). The Unit Problem in the Thermodynamic Calculation of Adsorption Using the Langmuir Equation. Chem. Eng. Commun..

[85] Kresse G., Furthmüller J. (1996). Efficient iterative schemes for ab initio total-energy calculations using a plane-wave basis set. Phys. Rev. B Condens. Matter Mater. Phys..

[86] Wang V., Xu N., Liu J.C., Tang G., Geng W.T. (2021). VASPKIT: A user-friendly interface facilitating high-throughput computing and analysis using VASP code. Comput. Phys. Commun..

[87] Sujith C.P., Joseph S., Mathew T., Mathew V. (2022). First-principles investigation of structural, electronic and optical properties of quasi-one-dimensional barium cadmium chalcogenides Ba_2_CdX_3_ (X = S, Se, Te) using HSE06 and GGA-PBE functionals. J. Phys. Chem. Solids.

[88] Perdew J.P., Burke K., Ernzerhof M. (1996). Generalized gradient approximation made simple. Phys. Rev. Lett..

[89] Kohn W., Sham L.J. (1965). Quantum density oscillations in an inhomogeneous electron gas. Phys. Rev..

[90] Monkhorst H.J., Pack J.D. (1976). Special points for Brillouin-zone integrations. Phys. Rev. B.

[91] Cherifi K., Cheknane A., Hilal H.S., Benghia A., Rahmoun K., Benyoucef B. (2020). Investigation of triphenylamine-based sensitizer characteristics and adsorption behavior onto ZnTiO_3_ perovskite (1 0 1) surfaces for dye-sensitized solar cells using first-principle calculation. Chem. Phys..

[92] Nor N.U.M., Mazalan E., Risko C., Crocker M., Amin N.A.S. (2022). Unveiling the structural, electronic, and optical effects of carbon-doping on multi-layer anatase TiO_2_ (1 0 1) and the impact on photocatalysis. Appl. Surf. Sci..

[93] Samanta P.K., English N.J. (2019). Opto-electronic properties of stable blue photosensitisers on a TiO_2_ anatase-101 surface for efficient dye-sensitised solar cells. Chem. Phys. Lett..

[94] Guo W., She Z., Yang S., Xue H., Zhang X. (2020). Understanding the influence of Lu, La and Ga active elements on the bonding properties of Sn/SiO_2_ interfaces from first principle calculations. Ceram. Int..

[95] Chang X., Li X., Xue Q. (2022). Sensing mechanism of acetone adsorption on charged ZnO and ZnSe surfaces: Insights from DFT calculations. Mater. Today Commun..

[96] Yang X., Yan Z., Gao G., Gao R., Zhang T., Su H., Tian M., Wang S. (2022). Understanding of Photocatalytic Partial Oxidation of Methanol to Methyl Formate on Surface Doped La(Ce)-TiO_2_: Experiment and Dft Calculation. SSRN Electron. J..

[97] Lai W., Zhang K., Shao P., Yang L., Ding L., Pavlostathis S.G., Shi H., Zou L., Liang D., Luo X. (2019). Optimization of adsorption configuration by DFT calculation for design of adsorbent: A case study of palladium ion-imprinted polymers. J. Hazard. Mater..

[98] Eskandari P., Farhadian M., Solaimany Nazar A.R., Goshadrou A. (2021). Cyanide adsorption on activated carbon impregnated with ZnO, Fe_2_O_3_, TiO_2_ nanometal oxides: A comparative study. Int. J. Environ. Sci. Technol..

[99] Bettoni M., Falcinelli S., Rol C., Rosi M., Sebastiani G.V. (2020). Gas-Phase TiO_2_ Photosensitized Mineralization of Some VOCs: Mechanistic Suggestions through a Langmuir–Hinshelwood Kinetic Approach. Catalysts.

[100] Shokry A., Khalil M., Ibrahim H., Soliman M., Ebrahim S. (2021). Acute toxicity assessment of polyaniline/Ag nanoparticles/graphene oxide quantum dots on Cypridopsis vidua and Artemia salina. Sci. Rep..

[101] Nunes B.S., Carvalho F.D., Guilhermino L.M., Van Stappen G. (2006). Use of the genus Artemia in ecotoxicity testing. Environ. Pollut..

[102] de Santana D.C.N., Perina F.C., Lourenço R.A., da Silva J., Moreira L.B., de Souza Abessa D.M. (2021). Levels of hydrocarbons and toxicity of water-soluble fractions of maritime fuels on neotropical invertebrates. Ecotoxicology.

[103] de Oliveira É.C., da Silva Bruckmann F., Schopf P.F., Viana A.R., Mortari S.R., Sagrillo M.R., de Vasconcellos N.J.S., da Silva Fernandes L., Bohn Rhoden C.R. (2022). In vitro and in vivo safety profile assessment of graphene oxide decorated with different concentrations of magnetite. J. Nanoparticle Res..

