# Supplementary material for: Cyanide Removal by ZnTiO3/TiO2/H2O2/UVB System: A Theoretical-Experimental Approach
Source: Int J Mol Sci. 2023 Nov 17;24(22):16446. doi: 10.3390/ijms242216446 (PMC10671060; doi:10.3390/ijms242216446)
Supplement: Supplementary file 1 [file ijms-24-16446-s001.zip › ijms-2678030-supplementary.pdf]

Supplementary Material

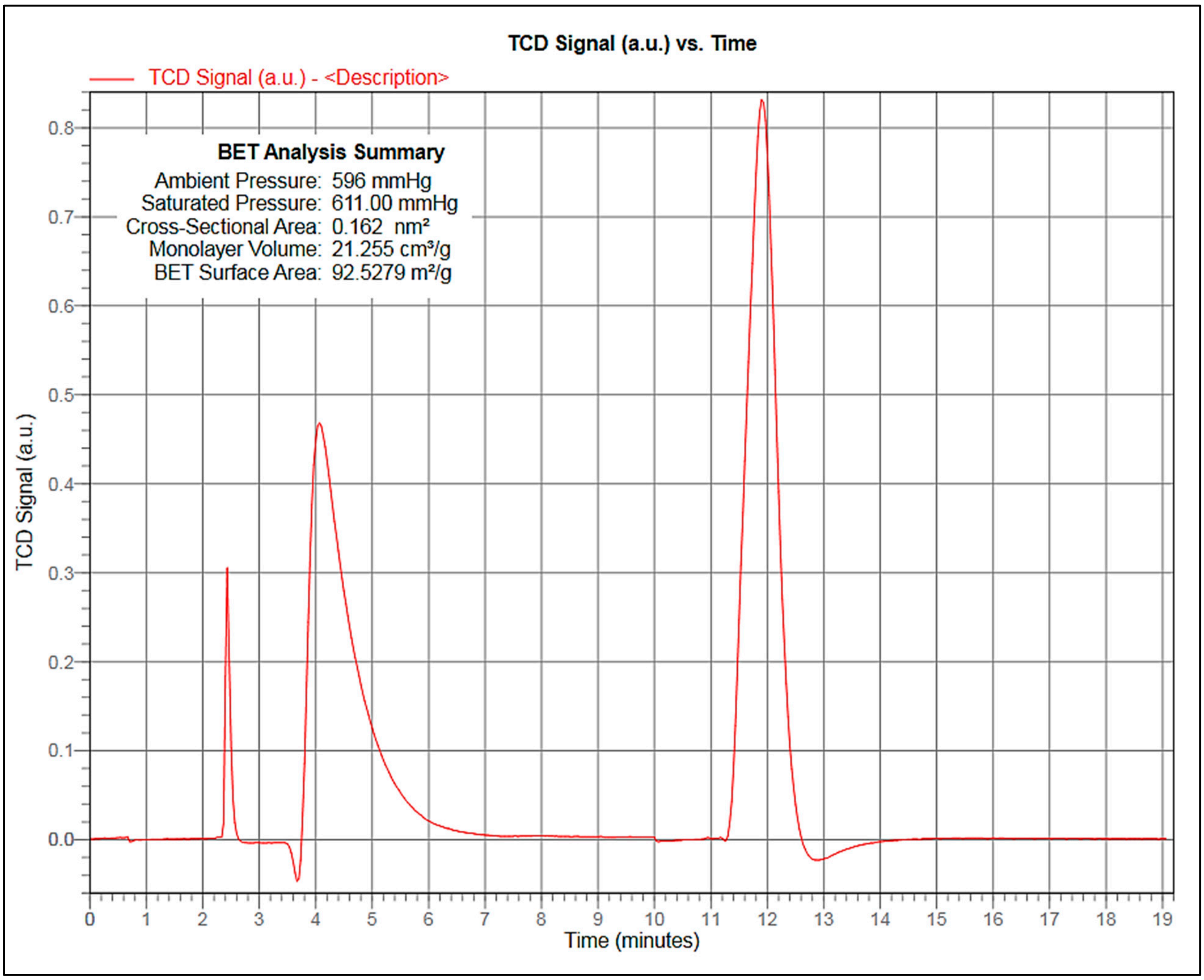

Figure S1. Plot of TCD Signal (a.u.) versus time from SSA analysis using the BET method.

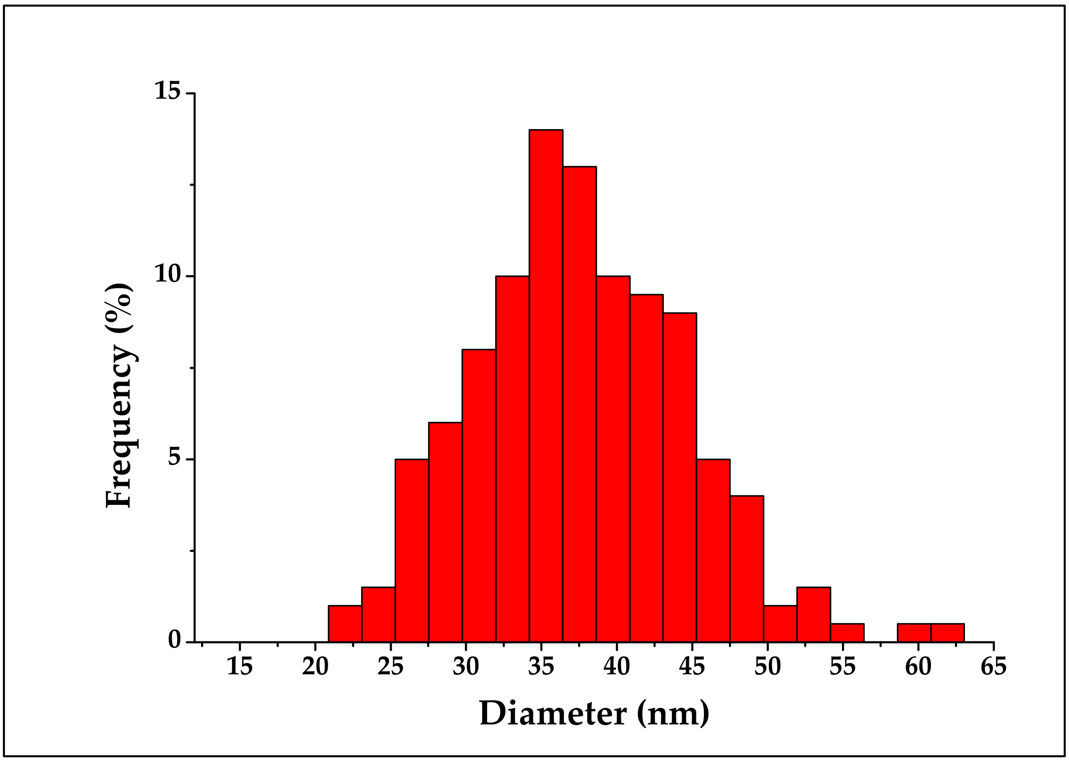

Figure S2. Particle size distribution of TiO_2 nanoparticles.

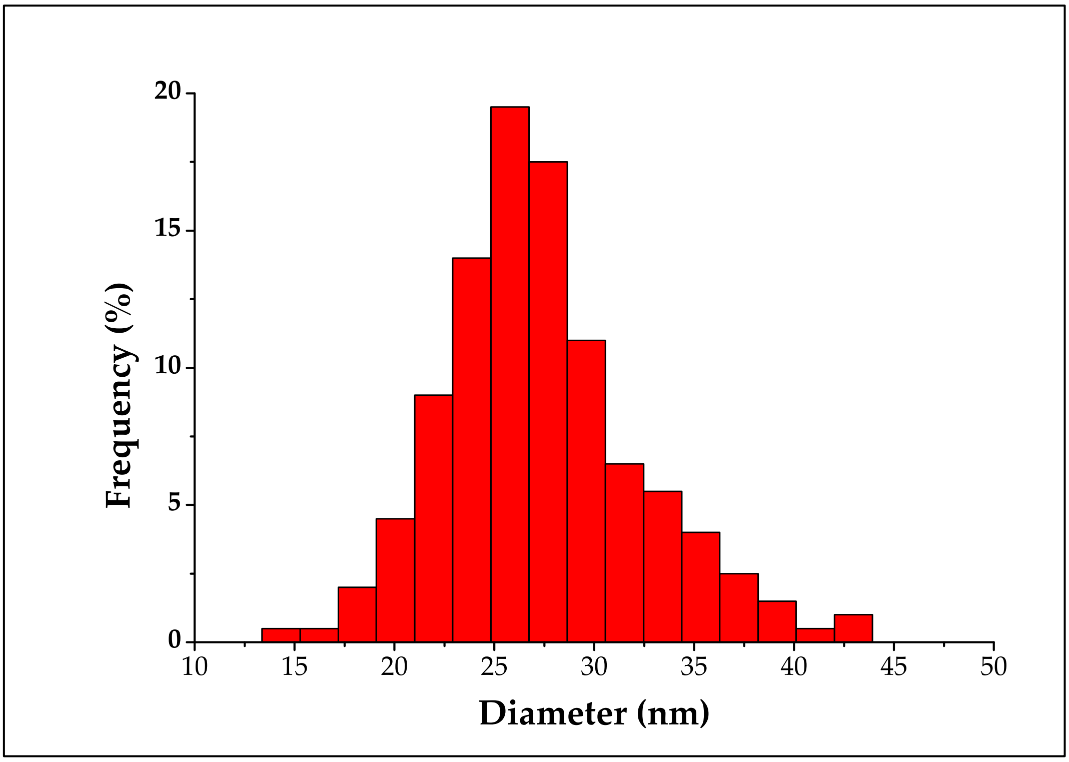

Figure S3. Particle size distribution of $ZnTiO_3/TiO_2$ nanoparticles.
